# Too much or too little information: how unknown uncertainty fuels time inconsistency

**DOI:** 10.1007/s43546-021-00189-9

**Published:** 2022-01-19

**Authors:** Inhwa Kim, Keith J. Gamble

**Affiliations:** 1 Department of Economics and International Business, Sam Houston State University, Huntsville, TX 77341-2118 USA; 2 Department of Economics and Finance, Jennings A. Jones College of Business, Middle Tennessee State University, Murfreesboro, TN 37129 USA

**Keywords:** Present bias, Risk preference, Intertemporal choices, Uncertainty, Information acquisition, C9, C11, D81, D83, G0, G4

## Abstract

Under uncertainty, there is considerable heterogeneity in expectations of results, and the outcome of each choice is a reflection of those expectations. This study aims to understand the role of subjective probabilistic inference in updating information for decision-making procedures under uncertainty. We show that adding uncertainty of trade-offs in decision-making criteria induces more inconsistent present preferences. We find that subjective probabilistic inference results in different levels of information acquisition, which plays a central role in many everyday cases of forecasting. The result of forecasting exerts substantive constraints on cognitive processes and shapes a type of restriction or stimulus in decision-making procedures. As uncertainty increases, generated fear of losses turns into an obstacle to the information acquisition process, and especially participants with low probabilistic inference tend to overestimate or underestimate future unknown rewards. In addition, our experiment shows that risk preference does not play a key role in decision-making procedures under unknown uncertainty. This finding is an experimental manifestation of Knight’s argument (Risk, uncertainty, and profit, Houghton Mifflin, Boston, 1921), which explains unknown uncertainty, and shows the relationship between cognitive ability and time inconsistency.

**Supplementary Information:**

The online version contains supplementary material available at 10.1007/s43546-021-00189-9.

## Introduction

This paper aims to understand the mechanism of time preferences in behavioral economics. Adding the uncertainty of trade-offs to decision-making criteria induces more inconsistent time preferences. Our motivation stems from theoretical and empirical work that demonstrates three closely related issues dealing with prospect theory: (1) greater cognitive ability, especially mathematical ability, is positively related to being more patient and less risk-averse (Benjamin et al. 2013); (2) increased risk decreases subjects’ patience levels (Anderson and Stafford 2009); (3) subjects with perfect foresight generate small discounts for future payoffs (Gabaix and Laibson 2017). Taken together, these three key ideas suggest that as an immediate or near-future reward is delayed, subjective probabilistic inference is related to decision-making procedures in intertemporal choices under uncertainty. An explanation of probabilities from a “subjective” point of view is “consciously and unconsciously in one’s mind”. This paper proposes a unique empirical model to help us understand which measures of cognitive skill might drive the cause and effect relationship in intertemporal choices and analyzes the results of 168 students’ intertemporal choices collected through the Psychology Department Research Pool at Middle Tennessee State University (MTSU).

One of the drawbacks of the most amazing phenomena in human consciousness is the ability to worry about the future. We know the future exists, but we don’t know what will happen in that future. Uncertainty itself induces many different consequences, especially for humans. The unique thing about humans is their ability to reflect on the fact that these future events are unknown or unpredictable. In ambiguous or unpredictable situations, many individuals look for clues in the environment or use what they know from past experiences to make their predictions. We focus especially on behavioral and cognitive inference among the characteristics of individuals used in the decision-making process.

“Statistical thinking will one day be as necessary for efficient citizenship as the ability to read and write,” said H. G. Wells (1903), author of the novel Time Machine. We argue in this paper that ignored or misinterpreted statistical information can affect the way individuals perceive and interact. We concentrate on the ability of some agents to make more informed decisions: subjective statistical inference. This ability is connected to information update ability. Heterogeneous choices are the result of different applications or adjustments of given information. Bayes’ rule is well known for updating probabilities as new information is acquired. It is to start with a percentage hypothesis, gather evidence, and update the hypothesis. “Evidence adjustment” represents how much participants use evidence after gaining information. Most information for choice problems contains or generates quantitative information, and from this quantitative information, agents can determine the probabilities for each reward. The ability to apply information varies from person to person. Generally, the average person does not naturally think in terms of the Bayes’ rule. Because odds compare the relative quantity or frequency of scenarios while percentages use a part-to-whole quantity or frequency, the concept of “percentage” creates an obstacle in understanding the problem rather than the concept of “ratio.” Kahneman and Tversky (1973, 1979, 1986) wrote that the base rate fallacy or base rate neglect is a tendency for people to misjudge the possibility of a situation by not considering all relevant data. This fallacy demonstrates how sensitive an individual is to accepting given information. In this paper, we use this fallacy to study how each agent’s subjective probabilistic inference affects the decisions of intertemporal choice. Probability is the exact belief found in the problem-solving procedures. We examine that even the Bayesian formula, which allows us to overcome the imperfections of faith and converge on the truth, cannot be helped in the face of preconceptions and prejudices of extremely biased individuals.

Uncertainty turns a given choice problem into a predictive one, such as a lottery or a roulette game. An agent has to predict uncertain rewards by given information or intuition. Each agent may use his or her statistical inference skill and reward expectations to guess the expected amount of compensation. While some agents rely more on statistical inference for a prediction, other agents put weight on their expectations for the value of rewards. An agent with sufficient foresight considers his intuition to be an appropriate substitution and replaces the formation of foresight with possible statistical inference. On the other hand, an agent with imperfect foresight relies more on her intuition for uncertain compensation. Gabaix and Laibson (2017) model myopia in the context of logical noise that causes the signal to be inaccurate for forecasts. We agree with Gabix and Laibson that myopic agents fail to handle an intertemporal trade-off carefully and show more discounting. Also, we expect that adding unknown uncertainty generates heterogeneous and inconsistent time preferences.

Savage (1954) presented the normative theory of choice under uncertainty. Savage presented axioms that guarantee the existence of pairs of probabilities and utility functions. He argued that this probability can be expressed as an individual’s preference maximizing expected utility. His axioms are similar to the axioms presented by Ramsey (1926), but provide more sufficient evidence in decision theory. Knight distinguished risk from uncertainty in his 1921 book Risk, Uncertainty and Benefit. According to Knight, risk applies to situations where the outcome of a given situation is unknown, but the probability can be measured accurately, while uncertainty applies to situations in which all the information needed to set the exact probability in the first place is unknown. This distinction between risk and uncertainty is meaningless in Savage’s theory because Savage applied all uncertainties as risks. This paper argues that uncertain but probabilistic information is given in the selection should be distinguished from uncertainty without any information. Uncertainty without information can induce inconsistent choices of individuals. Knight’s (1921) distinction between risk and uncertainty can help to analyze the behavior of individuals’ choices when unexpected uncertainty occurs and risk increases. In this paper, we subdivide uncertainty into uncertainty with information and uncertainty without information.

Through a hypothetical survey experiment, we examine the distribution of agents’ intertemporal choices when their rewards are clear or uncertain, respectively. In particular, we study the relationship between the expectations of each individual’s future unknown reward and their practical choices when uncertainty is maximized. This is an experimental manifestation of Knight’s argument (1921). We investigate the impact of risk preference on the distribution of choices, along with time preference. Each individual’s information acquisition ability leads to predict the expected value of future rewards based on the value of alternative rewards, which results in a heterogeneous choice under uncertainty. Even if uncertainty still exists, some individuals modify decision-making procedures through given information, while others ignore given information. Our first result highlights that when the appeal of immediate rewards has disappeared, the subjective probabilistic inference has a positive effect on intertemporal choices under known uncertainty, but not under unknown uncertainty. Known uncertainty here refers to when there is uncertainty in the choice criteria but additional information is given, and unknown uncertainty indicates when no information is given. The higher the subjective probabilistic inference, the higher the expectation of future utilities over time. We can interpret that forecasts through probabilistic inference affect an individual’s tolerance. This finding is consistent with the result described by Benjamin et al. (2013) who explain that greater cognitive ability, especially mathematical ability, is positively related with being more patient. Also, we find that good statistical knowledge and having taken a statistics class positively affect the attainment of strong information acquisition and especially on probabilistic inference. Agents with low subjective probabilistic inference tend to ignore information about future rewards. However, if the information is applied but the individual’s risk preference is clear, some choices may be contrary to the given information.

Another contribution of this paper is that we confirm that risk preference does not play an essential role in decision-making procedures under unknown uncertainty. Under known uncertainty, risk preferences, along with time preferences, play an important role in decision-making procedures. However, uncertainty without any information leads many individuals to refuse to wait more periods for unknown rewards. Even risk-seeking individuals who are optimistic about unknown future rewards also hesitate to choose to wait for more periods. When individuals face maximized uncertainty, fear of loss hinders the acquisition of information and makes some hesitate to apply it in decision-making procedures. Our result shows that uncertainty associated with risk preferences affects each individual’s choice, and also uncertainty itself affects decision-making procedures. This finding can be an expansion of the researches of Fréchette et al. (2017) and Golman and Loewenstein (2018). We also confirm that information is an important criterion of choice. When we provide catchable information in the experiment, participants with high subjective probabilistic inference tend to apply and adapt more of the hidden information of future rewards to their decision-making procedures. Even though no information was given, our data shows an anchoring phenomenon in which many participants applied the information given in their previous choices to their forecasts.

In the following section, we review the background of this study. “Model structure” outlines theoretical frameworks, “Research design” introduces the experimental research designs, and “Regression results” shows the regression results of lab experiments and compares ones with other models. The last section discusses conclusions and limitations.

## Background

Time preference studies have made many advances over the decades. O’Donoghue and Rabin (1999) introduce “present bias,” a tendency to give stronger weight to results closer to the current time when people consider the trade-off between two different time moments. Present-biased individuals stop their efforts to find the best choice too quickly, leading to actions that do not specifically consider, select, or re-evaluate the various conditions and outcomes across time. According to O’Donoghue and Rabin (2003), a naïve individual can be more affected by the present bias parameter, may exhibit dynamically contradictory behaviors, and have more self-control problems than others. Laibson (1997) explains that individuals have inconsistent time preferences. Some individuals place a disproportionately greater weight on short-term events than long-term. Loewenstein and Prelec (1992), and Benhabib et al. (2010) show that people do not have a constant discount rate over time. Weitzman (1998) presents the theoretical reasons why the future should be discounted at a lower rate. Andreoni and Sprenger (2012) introduce the Convex-Time Budget (CTB) approach to estimate the individual discount rate over a long period. They can simultaneously estimate time preference and utility by convexifying budgets. With reference to the passage of time, the asset-based approach has been successfully applied to money-related discount decisions (see, for example, Ericson et al. 2015; Leland 2004; Read et al. 2012; Rubinstein 2003; Scholten and Read 2010). Müller and Rau (2021) find that present-biased subjects involve panic buying during the COVID-19 pandemic crisis. They also show that risk tolerance negatively affects compliance to social distancing. Backes-Gellner et al. (2021) have presented empirical evidence that time preference and risk preference affect education and labor market entry decisions. Gabaix and Laibson (2017) prove theoretically that an agent’s prior choice distribution dominates the expectation of future utilities, and imperfect foresight creates similar outcomes with the classical time preferences. Our research provides further empirical tests of these theories.

Along with a number of theoretical developments in choice preferences, the past few decades have seen a debate over the impact of cognitive abilities on decision-making procedures. Among cognitive skills, statistical problem solving abilities vary systematically by gender and age, as well as cultural environment. Men, in general, outperform women, though the difference is not large. Benjamin et al. (2013) find that students who do better on standardized math tests are more patient and less risk-averse in a gambling experiment. A group of scholars documents the importance of general and specific cognitive skills for decision-making (see, for example, Benjamin et al. 2013; Frederick 2005; Peters et al. 2006; Stanovich and West 2008). According to these scholars, individuals with higher cognitive skills are relatively much less likely to buy at standard market prices over individuals’ market prices. Another group of papers shows in experimental studies, higher-level cognition improves risk assessment and decision-making skills (see, for example, de Bruin et al. 2007; Peters et al. 2006). Martins (2021) argues that people are generally considered to be poor at probabilistic reasoning in cognitive inference tasks and prone to irrational biases. Also, he has shown that the gap between perception and cognition is related to a subjective mechanism for processing information. To provide a methodological explanations for the differences in individual cognitive abilities, belief and information estimation tests using Bayesian formulations are increasingly used in perceptual and learning tasks. Hein et al. (2021) find that the temporary anxiety of individuals has become more resistant to Bayesian belief updates, ultimately leading to the impairment of reward-based learning. Steiner and Stewart (2016) show that Bayesian inference generates overweight of small probability events, as in prospect theory. Our experiments provide supporting evidence for the type of perceptual processes proposed in different scenarios of choices.

Causality between patience under uncertainty and various measurements of performance remains an open question. According to Kahneman and Tversky (1979), loss aversion posits that the disutility of losses exceeds the utility of corresponding gains. Many studies have examined gains or losses in experiments since the advent of the idea of loss aversion (see, for example, Ariely et al. 2005; Brenner et al. 2007; Inesi 2010; Kahneman and Tversky 1984; Kermer et al. 2006; Schmidt and Zank 2005). With regard to loss aversion, risk aversion is a general preference for safety and certainty over uncertainty, and for the possibility of loss or pains. Over the past few decades, the focus of economic research on behavior under uncertainty has shifted from risk aversion to show that low- or high-risk preferences affect many decisions. Risk-seeking investors tend to invest in high-risk assets, while risk-averse investors avoid investing in high-risk assets (Corter and Chen 2006; Nguyen et al. 2016; Mahmoud and Pak 2015). Ainia et al. (2019) have presented that a risk-seeking attitude and overconfidence take important parts of investment decision making, while loss aversion does not affect investment decision making. Another consistent research finding is that prudence and temperance play especially important roles in saving decisions (see, for example, Eeckhoudt 2002 (medical decision making); Embrey et al. 2015 (bargaining); Esö and White 2004 (auctions); Felder and Mayrhofer 2014 (medical decision making); Kocher et al. 2015 (auctions)). Falk et al. (2018) and Potrafke (2019) both find that patience and intelligence have a positive correlation. Other interesting research argues that risk aversion increases with age (see, for example, Boyle et al. 2012; Brooks et al. 2018; Bucciol and Miniaci 2011; Jianakoplos and Bernasek 2006). This argument is also in line with Gabaix and Laibson’s (2017) finding that there is a decrease in present-biased time preferences due to increased human experience. Understanding how the interaction of individual cognitive abilities and risk preferences affects intertemporal choices is a significant question.

Studies of uncertainty in various fields, including financial market research through human emotion, behavior, and belief, are being conducted actively. Knight’s theory against the dominant claims of uncertainty and risk causality in social studies was published in 1921. He argues that uncertainty and risk should be recognized as the respective factors that influence decision making. Many arguments for and against Knightian uncertainty have been continued. A large body of empirical research shows support for Knight’s unknown uncertainty by delineating ambiguous and unambiguous options (see, for example, Chow and Sarin 2002; Fox and Weber 2002; Roca et al. 2006). In 2017, Frechette, Schotter, and Trevino show that in uncertain environments where information about unknown probability distributions can be obtained, personality variables influence their choice. Golman and Loewenstein (2018) present a theory of preferences of information based not only on information gap but also on the thoughts and feelings of information by decision-makers. They explain that material rewards, faith, and attention play important roles in measuring utility for decision-making procedures. Studies that are not in favor of Knightian uncertainty argue that it is not easy to properly implement the uncertainty context in field experiments (see, for example, Welter et al. 2016). Brooke and Cheung (2021) deliver that Knight overlooked the form of partial information in the distinction between risk and uncertainty. We hypothesize that known and unknown uncertainties can induce different decisions in intertemporal choices. We carefully design the provision of catchable information under uncertainty and present it to participants. Our research provides further insights in this direction.

We have seen that uncertainty and risk are connected but are not the same. Many studies show that uncertainty and risk affect decision-making procedures. We make a novel contribution to the literature by integrating statistical inferences and choice preferences into a single framework and exploring the role of risk preference and present bias in intertemporal choices.

## Model structure

A long-term choice utility will be fewer than current utilities and the reduced utility varies from person to person. Each person has a different analysis of information and they react differently. Adding uncertainty to the time-dependent utility will result in an even more heterogenous set of individual utilities. Individuals’ various choices are the result of different applications of uncertain information. What we would like to have is an adequate measurement that can mirror a subjective information acquisition ability. We hypothesize that a subject’s base rate fallacy level is related to her ability to aggregate information that can affect different time-based payoff decision procedures.

### Choice preference

To explore the implications of accepting and adjusting new information on the decision-making process which can reveal individuals’ time preferences, we consider two different models of the simplest of intertemporal choices: earlier payoff and later but larger payoff. We follow the beta-delta model by Laibson (1997) and the foresight-model by Gabaix and Laibson (2017). The main outcomes of these models relate to maximizing utility of individual *i*. We start by considering the expected utility model: the total utility from the current period *t* is

$$\begin{aligned} U_{t}=\sum _{K=0}^{T}D\left( t+K\right) \cdot u\left( r_{t+K}\right) , \end{aligned}$$

where *T* is the last period of choices, $u\left( \cdot \right)$ is an instantaneous utility, $r_{t+K}$ is a reward in period $t+K$ ($K\in {\mathbb {N}}\cup \left\{ 0\right\}$), and $D\left( K\right)$ represents the discount function factor with $D\left( 0\right) =0$ and $D^{'}\left( t\right) \le 0$ such as

$$\begin{aligned} D\left( t\right) ={\left\{ \begin{array}{ll} \beta \delta ^{t}, &{} t\in N^{+}\,\text { and }\,0\leqq \beta <1\\ 1, &{} t=0 \end{array}\right. }. \end{aligned}$$

Here, the parameter $\beta$ represents a present preference and $\delta$ represents a future utility discount factor. We also assume that the reward for a long-term choice is greater than or equal to the reward for a short-term choice. That means, instantaneous utilities between short-term and long-term satisfy $u\left( r_{t}\right) \le u\left( r_{t+K}\right)$.

A subject chooses *Early* if and only if ${\displaystyle D\left( t\right) u}\left( r_{t}\right) >D\left( t+K\right) u\left( r_{t+K}\right)$. The opposite case is defined as an agent chooses *Late*. To analyze the role of time preference, we consider two types of people representing extreme assumptions about such preferences: *present* *biased* *agents* prefer *Early* rewards to *Late* rewards and *non*-*present* *biased* *agents* prefer *Late* rewards to *Early* rewards. We consider the intertemporal utilities of each agent’s choices as a normal distribution.

### Level of base rate fallacy

Bayesian statistics is used as a special form of statistical inference applied to the analysis of experimental data in many real situations. These statistical methods are designed to contribute to the process of making scientific judgments in the face of uncertainty and variation. Bayes’ rule provides a way to revise and update the existing predictions of a hypothesis when there is new or additional evidence, leveraging the conditional probability. Considering a hypothesis *H* and evidence *E*, Bayes’ rule states that the relationship between the hypothesis probability before obtaining evidence *P*(*H*) and the hypothesis probability after obtaining evidence $P(H\vert E)$ is

$$\begin{aligned} P(H\vert E)=\frac{ P(E\vert H)P\left( H\right) }{P(E\vert H)P\left( H\right) +P(E\vert H^{C})P\left( H^{C}\right) }=\frac{P(E\vert H)P\left( H\right) }{P\left( E\right) }. \end{aligned}$$

Here, we note that $$P\left( H\right)$$ is called the prior probability which is the possibility of the event under the given information, $$P(H\vert E)$$ is called the posterior probability, and $$L=\frac{P(E\vert H)}{P\left( E\right) }$$ is called the likelihood. Bayes’ rule is to start with a percentage hypothesis, gather evidence and update the hypothesis. It can be used to predict how the probability of an event occurring is affected by the hypothetical new information, assuming that the new information will be found to be true. For example, we assume that an agent shows *z*% of prior probability of willingness to wait in intertemporal choices between $*r* today and $*R* in six months where $$r<R$$: $$P(H)=P(\text {willingness to wait})=z$$%. If we suggest him additional information on the next selection: $*r* today and 50% chance of getting a big reward $2*R* in six months, then the new probability of willingness to wait is

$$\begin{aligned} \begin{aligned} P(H\vert E) = P(\text {willingness to wait} \vert 50\% \ \text {chance of getting a big reward in 6 months}), \end{aligned} \end{aligned}$$

which is the posterior probability. Here, the expected value of getting a big reward in six months is same as the previously suggested amount of $*R*. The probability of evidence as well as the prior probability has a key role in the posterior probability. It is important that information update does not require new principles or special inference as a result of probability theory. This paper focuses on the base rate fallacy from Bayes’ rule which is one of the cornerstones of Bayesian statistics. It is a tendency to determine the probability of an event based on unrelated information rather than on the probability of actual evidence. We investigate how accurately each participant applies the new additional information given to their choices through the base rate fallacy. Some agents focus on whether the evidence is relevant to the prior or not, and they decide how much weight to put on the previous experiences. We are interested in the level of “evidence adjustment” of each agent and we apply the weighting probabilities (Fox and Poldrack 2008; Tversky and Kahneman 1992) instead of original hypothesis probabilities as follows:

$$\begin{aligned} \begin{aligned}&\left\{ P\left( H \right) , P\left( H^{c}\right) \right\} = \left\{ \frac{\left( P\left( H\right) \right) ^{1-\alpha }}{\left( \left( P\left( H \right) \right) ^{1-\alpha }+\left( P\left( H^{c}\right) \right) ^{1-\alpha } \right) ^{\frac{1}{1-\alpha }}}, \frac{\left( P\left( H^{c}\right) \right) ^{1-\alpha }}{\left( \left( P\left( H \right) \right) ^{1-\alpha }+\left( P\left( H^{c}\right) \right) ^{1-\alpha } \right) ^{\frac{1}{1-\alpha }}}\right\} . \end{aligned} \end{aligned}$$

Then as a mathematical formula, the base rate fallacy is defined by

1 $$\begin{aligned} P_{\alpha }(H\vert E)=\frac{P(E\vert H)\left( P\left( H \right) \right) ^{1- \alpha }}{P(E\vert H)\left( P\left( H\right) \right) ^{1-\alpha }+P(E\vert H^{\text {c}})\left( P\left( H^{c}\right) \right) ^{1-\alpha }} \end{aligned}$$

where $\alpha \in \left[ 0,1\right]$ is the level of base rate fallacy. This equation explains that agents with high level of base rate fallacy ignore given information more than agents with low level of base rate fallacy.

To compute the parameter $\alpha$, we use the classic scenario introduced by Kahneman and Tversky (1972): the “Cab Driver Problem.” Subjects are asked to answer this problem, and from their answers, we can calculate how strongly or weakly each agent ignores the Bayes’ rule.

Kahneman and Tversky’s example “A cab was involved in a hit-and-run accident at night. Two cap companies, the Green and the Blue, operate in the city. Eighty-five percent of the cabs in the city are Green and fifteen percent are Blue. A witness identified the cab as Blue. The court tested the reliability of the witness under the circumstances that existed on the night of the accident and concluded that the witness correctly identified each one of the two colors 80% of the time and failed 20% of the time. What is the probability that the cab involved in the accident was Blue rather than Green?”

Using Bayes’ theorem to solve the problem, we set the hypothesis *H*: Accident caused by Blue cab, and the evidence *E*: Witness said the cab was Blue. Using the Bayes’ rule,

$$\begin{aligned} P(H\vert E)=\frac{P(E\vert H)P\left( H\right) }{P\left( E\right) }=\frac{0.8\times 0.15}{0.8\times 0.15+0.2\times 0.85}\thickapprox 0.41. \end{aligned}$$

If an agent answers this question with $x\in \left[ 0,1\right]$, then we can draw his level of base rate fallacy, $\alpha$, using Equation (1) as follows:

$$\begin{aligned} \frac{0.8\times \left( 0.15\right) ^{1-\alpha }}{0.8\times \left( 0.15 \right) ^{1-\alpha }+0.2\times \left( 0.85\right) ^{1-\alpha }}=x. \end{aligned}$$

We expect that her answer is between 0.41 and 0.80. When the agent can use Bayes’ rule perfectly in their decision-making process, then her answer is 0.41. In cases where the agent fails to apply Bayes’ rule, we expect her answer is close to 0.80. If a participant’s answer does not fall between 0.41 and 0.80, we consider her level of base rate fallacy to be 1 where subjective probabilistic inference is lowest.

Since the time preference parameter is related to intra-personal decision problems, we consider that a different level of ignorance of Bayes’ rule. We hypothesize that the level of base rate fallacy affects the ability of each agent to solve the given problem based on total information. If an agent ignores Bayes’ rule in conditional statistics questions, then she fails to accept the whole value of given information in the decision procedures. Base rate fallacy affects each subject’s ability to update information, and it leads to heterogenous choice outcomes. We also question whether subjective probabilistic inference works in the individual decision-making procedures when no information is provided for the choice.

### Type of risk

Uncertainty creates risk into choices and is directly linked to a tolerance of waiting periods. When individuals are aware of risks, the degree of utility for each individual’s choice depends on their risk preferences along with the expected value of rewards. We can categorize each subject as risk-neutral, risk-aversion, or risk-seeking.

#### Definition 1

We compute that there exists the expected utility of each agent’s choice as ${\mathbb {E}}\left[ u\left( {\text {choice}}\right) \right]$ and the utility of expected value of their choices as $u\left( {\mathbb {E}}\left[ {\text {choice}}\right] \right)$. Then,

1. Agents with ${\mathbb {E}}\left[ u\left( {\text {choice}}\right) \right] <u\left( {\mathbb {E}}\left[ {\text {choice}}\right] \right)$ are risk-averse.
2. Agents with ${\mathbb {E}}\left[ u\left( {\text {choice}}\right) \right] =u\left( {\mathbb {E}}\left[ {\text {choice}}\right] \right)$ are risk-neutral.
3. Agents with ${\mathbb {E}}\left[ u\left( {\text {choice}}\right) \right] >u\left( {\mathbb {E}}\left[ {\text {choice}}\right] \right)$ are risk-seeking.

Research on the stability of risk preferences is central to microeconomics, but there have been many debates. A case in point is that individuals become more risk averse over their life cycle. Campbell and Shiller (1989) and Cochrane (2011) show that investors are more risk-averse during recessions than during booms. In 2018, Schildberg–Hörisch provide evidences of the long-term changes in risk preferences. We hypothesize that these changes are related to each individual’s information acquisition ability. Gabaix and Laibson (2017) argue that an agent’s prior choice distribution dominates the expectation for future utilities, and imperfect foresight creates similar outcomes with the classical time preferences. We also demonstrate that predicting unknown far-future rewards is a combination of each agents’ expectation for the future and his information updating ability. Information updating ability affects the degree of foresight which results in risk preferences. With regard to behavioral research of time preference, our results show how risk preferences work on intertemporal choice under unknown uncertainty. Furthermore, we would like to compare which characteristics of the decision maker interact with risk preference under unknown uncertainty to those under known uncertainty. This research contributes to both behavioral understanding of time preference and general studies of risk.

### Research hypothesis

We intend to proceed by comparing the effects of subjective probabilistic inference and different type of risk preferences in decision-making procedures in various choice situations. Here are our research hypotheses:

#### Hypothesis 1

Subjective probabilistic inference is the one of main factors in decision-making procedures in intertemporal choices without uncertainty.

#### Hypothesis 2

If probabilistic uncertainty is added to the intertemporal choices, subjective probabilistic inference and risk preferences steadily influence intertemporal choices.

#### Hypothesis 3

When maximizing uncertainty, risk preferences do not affect intertemporal choices.

#### Hypothesis 4

If uncertainty is maximized but outcomes from others’ previous choices are provided, subjective probabilistic inference and risk preferences both affect forecasting unknown future rewards.

In the following section, we introduce our research design and methodology.

## Research design

In this paper, we try to shed light on the subjective characteristics affecting each individual’s decision-making in different intertemporal choices. Kahneman (2011) argues that Bayesian inference tasks are generally solved by the representative experience formed by the intuitive and rapid processing System 1. Mandel (2007) explains that in the Bayesian Inference Task, people have problems adopting System 2 and naturally deduce the results using System 1, which is relatively prone to errors. Following previous research on biases in decision-making, we assume that when people make their intertemporal choice decisions, the phenomenon of the base rate fallacy will affect them. We also hypothesize that it is related to information acquisition ability. Even in simple moments of choice, people sometimes decide their choices based on probabilities to achieve more benefits or utilities. The value judgment is either rational inference or reflects the satisfaction of individual experiences.

Our analysis is built upon a unique lab experiment that focused on the ability to accept and apply information. The survey data provide us with information on demographic and intertemporal choices related variables: it enables us to estimate behavioral parameters, including subjective statistical inference, loss aversion, and present bias. In our experiment, we decide to discuss only the numerical dollar value of rewards, which is not completely generalizable to the psychological value of rewards or to other measures.

We use two primary dependent variables. The first dependent variable $y_{i}=\nabla {\text {WW}}_{i}$ is the change in number of willingness to wait as delays increase:

2 $$\nabla {\text {WW}}_{i} =\frac{ \# \; \text {of wait in near-future vs. far-future} - \# \; \text{of wait in immediate vs. far-future}}{\# \; \text {of wait in immediate vs. far-future} + 1}.$$

When $\nabla {\text {WW}}_{i}>0$, people are more willing to wait as delays increase. The larger the $\nabla {\text {WW}}_{i}$, the respondent’s willingness to wait is stronger.

Our second dependent variable, $\triangle {\text {MRS}}={\text {MRS}}_{2}-{\text {MRS}}_{1}$, is the difference in marginal rate of substitution. Here, ${\text {MRS}}_{i}$ is the marginal rate of substitutions between two rewards such as

$$\begin{aligned} {\text {MRS}}_{i}={\left\{ \begin{array}{ll} \beta \delta ^{t}\frac{u'\left( {\text {Late}}_{1}\right) }{u'\left( {\text {Early}}\right) } &{} \text {for }i=1~\left( \text {immediate vs. far-future}\right) \\ \delta ^{t-t_{1}}\frac{u'\left( {\text {Late}}_{2}\right) }{u'\left( {\text {Early}}\right) } &{} \text {for }i=2~\left( \text {near-future vs. far-future} \right) \end{array}\right. } \end{aligned}$$

where $t_{1}$ is the delay period of “immediate reward” and *t* is the “far-future reward” payment timing. Here, MRS shows trade-offs that the participants would be willing to make between the choices. When the appeal of immediate reward is disappear, this difference can reveal agents’ patience in intertemporal choices: the participant is patient when the $\triangle {\text {MRS}}$ is positive, but the participant is less patient when the opposite is true. Here, we use different utility functions: $u\left( x\right) =\sqrt{x}$ as a risk-averse; $u\left( x\right) =x^{2}$ as a risk-seeking; and $u\left( x\right) =x$ as a risk-neutral utility function. Although it is difficult to find the appropriate values of $\beta$ and $\delta$ for discordant choices, this choice indicates that participants further discount future utility when delaying initial early payments. We define $\triangle {\text {MRS}}=-\infty$ for the discordant choices.

### Basic experiment

#### Participants

While the theoretical motivation of this research has been briefly outlined above, the primary phase of this research will be focused on collecting and processing data. In the study to be described, our experimental starting point is observing each individual’s choice propensity. The experiments were conducted voluntarily in 2019 among students at MTSU. Using the Psychology Department Research Pool at MTSU, we collected data. The respondents were assured of anonymity. Participants were informed of the expected period of research, received written instructions, received information on data usage, and provided informed consent. They were only allowed to participate in the first page of the study if they were at least 18 years old, and we did not allow re-engagement in the online survey. These eligibility criteria were used in all our research. They were also informed about the reward for their participation in the research. Before participants were asked to complete the selection task, they were given a description of how to complete the selection task described in the example and were given detailed information on the meaning of all attributes and levels. This study is approved by the Institutional Review Board of MTSU. Datasets can be made available as supplementary material files, which will be accessible on the SN Business and Economics website upon publication.

We offer participants several types of intertemporal choice events: an Early payoff and a Late payoff. An Early reward $r_{t}$ will be provided after *t* time periods, while a Late reward $r_{t+K}$ will be provided after $t+K$ time periods. Independently, we elicit time preferences through a survey with statistical questions to estimate the level of the base rate fallacy. We examine whether each individual’s time preference parameters, $\beta$ and $\delta$, are different depending on the level of the base rate fallacy.

#### Summary statistics

Altogether 210 questionnaires were collected, but the final sample consisted of the answers of 168 participants due to extensively missing data. First, we collect demographic information and statistical knowledge of participants.

***Demographics***   Participants recorded their age, race, gender, marital status, size of a family, residency, occupations, education level, and income level. They also reported ownership of saving accounts, CDs, a retirement plan (for example, 401K plan), auto-loans, student-loans, or mortgage debts.

***Statistical knowledge***   Participants answered questionaries that are related to statistics. They answered some basic statistical questions and conditional probability questions.

ESM Table 1 presents the descriptive statistics of participants. There is a good balance of men and women in the group of respondents (Men: 48.81%). Regarding age level, a large number of our respondents fall into the 18 to 22 year age range. Of the 168 participants, 36.90% are in mathematics, science, engineering, and technology (STEM) majors; 29.17% are in business majors; 9.52% are in arts-related majors; 11.90% are in literature and social science majors; 12.50% are in other majors. Over fifty-two percent (52.38%) of the participants have taken or are taking statistics. A majority of participants have saving accounts. Over twenty-one percent (21.43%) of the participants have auto loans, and 46.43% of participants have student loans. Finally, the level of statistical knowledge ranged from 0 to 1. A level of $\leqq 0.25$ is set for poor- and $\geqq 0.75$ for good statistical knowledge. Almost thirty-eight percent (38.69%) of the participants have poor statistical knowledge, whereas 20.83% of the participants possess good statistical knowledge.

#### Measured the level of base rate fallacy

As mentioned earlier in “Level of base rate fallacy”, to establish a level of base rate neglect of each participant, we provide several questions that are designed in line with the “Cab Problem” (Kahneman and Tversky 1972). Our primary measure of the level of base rate fallacy $\alpha$ for each participant is the average response of the corresponding items on the questionnaire.

*Level of the base rate fallacy*   Participants answered the questionnaires that are related to base rate fallacy.

By measuring the individual judgment error determined by the difference between subjective and objective probabilities from Eq. (1), we can compute one of our independent variables, probabilistic inference as follows:

$$\begin{aligned} \text {probabilistic inference}=1-\text {a level of the base-rate fallacy}=1-\alpha . \end{aligned}$$

**Fig. 1:**
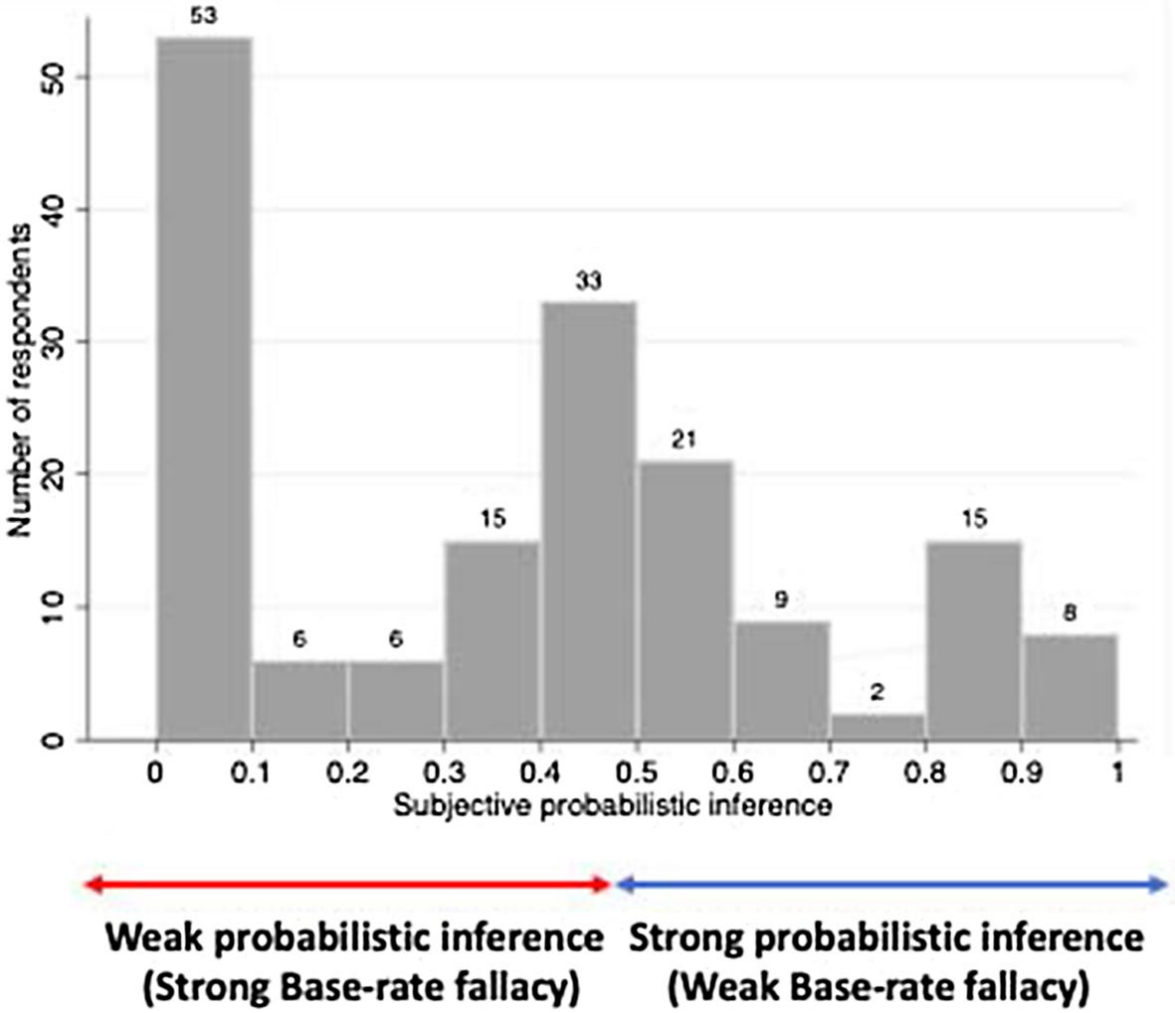
Subjective probabilistic inference

When participants can apply Bayesian rule perfectly in their decision-making process, their probabilistic inference is close to 1. When participants completely fail to account for Bayesian rule, their probabilistic inference is close to 0. We determine the entire range of 0 to 50 percent as ‘strong base rate fallacy’ and the rest as ‘weak base rate fallacy.’ Figure 1 shows the frequency distribution of 168 participants’ probability inference. This histogram reveals that 53 participants did not consider Bayesian rule at all, while eight participants correctly used it. Only a small number of participants understand the given problem situation and answer correctly. Fifty-eight out of 168 participants (34.52%) have a weak base rate fallacy, while the majority of participants (65.48%) have a strong base rate fallacy. ESM Table 2 shows that by gender, 29.82% of men and 39.86% of women tend to have “strong probabilistic inference,” while 18.29% of men and 15.11% of women have good statistical knowledge. In terms of probabilistic inference, the mean for men is 0.3188 and the mean for women is 0.4226. On the other hand, in terms of statistical knowledge, ESM Table 3 shows that the mean for men is 0.4482 and the mean for women is 0.4331. We can capture that while gender differences are not large in terms of statistical knowledge, many women have better probabilistic inference than men. Participants in STEM majors tend to be less prone to “base rate fallacy” than those in other majors. Taking a statistics class has a positive effect on the establishment of individual statistical knowledge and probabilistic inference.

### Experiment procedures

As mentioned in the introduction, this section explores the role of subjective probabilistic inference and present bias in intertemporal choice problems, which is characterized by two experimental structures: (1) early or later payoffs under the known amount of rewards, and (2) early or later payoffs under the unknown amount of rewards. Through the experiments, we can calculate two parameters: discounted future utility factor, $\delta$, and time preference parameter, $\beta$.

In the first experiment, all participants respond to two “certain” intertemporal choice scenarios. They are instructed that each trial is not related to the other. On each trial, participants choose one of two options. Each participant completes five following trials of each task: “Receive $1000 today or $X in 1 year: X = 1020; 1100; 1500; 2000; 10,000.” The second “certain” intertemporal choice scenario is between near- and far-future conditions: “Receive $1000 in 6 month or $X in 1 year: X = 1020; 1100; 1500; 2000; 10,000.”

In the second experiment, all participants respond to four “uncertain” intertemporal choice scenarios. The first scenario is about the “certain” amount of immediate payoff versus the “uncertain” amount of future payoff choice problems: “Receive $1000 today or 50% chance of receiving $X in 1 year: X = 2040; 2200; 3000; 4000; 20,000.” The future options are twice as large, but the probability of gains is only 50%. Thus, the expected value of the far-future options is the same as in Experiment 1. They also answer the scenario of near- versus far-future conditions. The following scenario is about the “certain” amount of immediate payoff versus the “uncertain” amount with a guaranteed minimum, $1000, of future payoff choice problems. The difference between the previous scenario is that all participants can be compensated at least with the same amount of immediate payoff when they are willing to wait a year. The expected value of the far-future options is the same as in Experiment 1. They also choose to choices in the scenario of near- versus far-future conditions with a guaranteed minimum.

The final experiment explores the changing choices of participants by maximizing uncertainty which is consisted of two different scenarios. This task procedure is similar to that used in Experiment 1 and 2, but we maximize uncertainty in choice conditions. In Experiment 3, participants are informed of the “certain” amount of *Early* reward and “unknown” amount of *Late* reward without any information. The first scenario in Experiment 3 is elicited choice preferences with a hidden *Late* reward. At the beginning of this experiment, all participants choose one of two options: “Receive $1000 today or a sealed envelope with an unknown amount of money inside in a year. They also complete a similar scenario in different payoff setup time frames: near- versus far-future. In this experiment, we investigate how the risk preference actually affects the time preferred choices. The next scenario is related to Bayesian updating procedures. Participants are given other people’s choice outcomes, and then they respond to the same problem set-ups as in the first scenario. They have next information: The last six people to be given a sealed envelope in one year discovered the following amounts inside their envelope “$0; $0; $5000; $5000; $0; $5000.” Then they can choose one of the following two options: “Receive $1000 today or a sealed envelope with an unknown amount of money inside in a year.” The last scenario is about near- versus far-future conditions. Compared to Experiment 2, participants forecast the amount of *Late* reward with others’ choice outcomes. Each forecast is a combination of expectations of future returns and information acquisition from others’ outcomes. We expect that this experiment could reveal the relationship between uncertainty and subjective future forecasting capabilities.

### Without uncertainty: near- vs. far-future

From these experiments, we can calculate two dependent variables: (1) discount factor, $$\delta$$, and (2) time preference parameter, $$\beta$$ by estimating reference points. But there is an ordinary problem. We cannot estimate two parameters properly in some cases: a participant is originally willing to wait a year for a big reward rather than choosing a small and immediate reward, but he changes his choice when delays increase. For example, one participant, who chooses to wait a year for over $1500 amount of reward instead of accepting $1000 reward immediately, opts not to wait a year for any amount of rewards when the initial reward of $1000 is delayed. ESM Table 4 leads us to better comprehend that the utility of time and money is different in intertemporal choice. On the side of traditional economics, when the appeal of immediate compensation disappears, the patience of participants will increase or be the same as before. But in the above case, she has a small value, almost close to 0, of the discounted future utility factor, $$\delta$$, but a huge value of the present preference parameter, $$\beta$$. The beta-delta model delivers the range of the discount factor: $$0\leqq \delta \leqq 1$$. Also, it is generally possible to use $$0\leqq \beta \leqq 1$$ to identify how discounting occurs when today is involved (Laibson 1997; Harrison et al. 2007). The above choice example does not follow this property. When $$\beta >1$$, we assume this type of choice is the outcome of contradictory decision-making. This inconsistency in intertemporal choices reveals that some participants lack the ability to predict about future utilities or cannot properly adjust given information to their choices. To solve this problem, we make a binary variable: *rationality* $$=0$$ for $$0\leqq \beta \leqq 1$$ and *rationality* $$=1$$ for $$\beta >1$$. Using this variable, we explore the characteristics of participants who make discordant choices. According to ESM Table 5, 26 out of 168 participants make discordant choices when there is no uncertainty in choice criteria. More than nineteen percent of participants in the weak probabilistic inference group and 8.62% of participants in the strong probabilistic inference group make discordant choices. There is not much difference in probabilistic inference between those who made a rational choice and an discordant choice among participants with strong probabilistic inference. However, among participants with weak probabilistic inference, the average probabilistic inference of participants who made rational choices is higher than those who made discordant choices.

ESM Table 2 shows participants’ intertemporal choices vary by uncertainty and subjective probabilistic inference. This leads us to focus on the endogeneity of probabilistic inference. We estimate an ordinary least square (OLS) regression in the following manner:

3 $$\begin{aligned} y_{i}=\rho _{0}+\rho _{1}probinf_{i}+\rho _{2}stat_{i}+\rho _{j}X_{i,j}^{'} +\varepsilon _{i}, \end{aligned}$$

where $probinf_{i}$ is a probabilistic inference parameter for individual *i*, $stat_{i}$ is a level of statistical knowledge, and $X_{i,j}^{'}$ is a demographic factor vector of covariates of degree *j*, including gender, age, major, owned financial items, and having taken statistical courses. The error term $\varepsilon _{i}$ is assumed to be normally distributed: $\varepsilon _{i}\sim N\left( 0,\delta _{\varepsilon }\right)$. The results for $\rho _{1}$ and $\rho _{2}$ in this OLS regression specified in Eq. (3) are presented in ESM Table 9.

### Under known uncertainty: non-guaranteed vs. guaranteed future minimum

There are no widely shared assumptions about the impact of risk preference on cognitive abilities or individual choices. Over the past few decades, there has been a heated debate over the links between cognitive abilities and risk preferences. A group of researchers insists that there is a positive link between cognitive abilities and risk preference: Donkers et al. (2001), Benjamin et al. (2013), Harrison et al. (2007), Hardeweg et al. (2013) and Jung (2015). Other researchers explain that there is a negative link: Hartog et al. (2002) and Hryshko et al. (2011). In this paper, we concentrate on the subjective probabilistic inference as a measure of the cognitive abilities.

To assess the relationship between subjective probabilistic judgment abilities and risk tolerances under uncertainty, Experiment 2 consists of two parts and each part consists of two scenarios: “under uncertainty without a guaranteed minimum” and “under uncertainty with a guaranteed minimum.” The expected values of the far-future options in both scenarios in Experiment 2 are the same as that of Experiment 1. Although the expected value of reward for the far-future is the same as for the previous experiment, participants feel more risk under the scenario of part one. Some participants choose based on the probability that they have a zero payoff, while others decide by focusing more on the size of the rewards they can obtain.

We first compare the number of rational and discordant choices by subjective probabilistic inference in conjunction with providing the guaranteed minimum. Compared to the total number of discordant choices in Experiment 1, ESM Table 5 shows that both percentage numbers of discordant choices under uncertainty are small. We expect participants to make more discordant choices under uncertainty, but our experimental results indicate the opposite. We understand that uncertainty in future rewards derives risk from intertemporal choices, and it reduces discordant choices by making participants more focused on the choice criteria. In particular, participants with strong probabilistic inference do not make discordant choices when there is a possibility that there will be no benefit to compensation in the far-future in their choices. When guaranteed minimum information is given, participants with weak probabilistic inference also make less unreasonable choices.

The next OLS regression measures the effect of uncertainty on risk preference. We can capture the effects by comparing two different scenarios: immediate versus far-future payoffs: and near- versus far-future payoffs. As uncertainty creates risk-preferences, we divide participants into three groups by their risk preferences: risk-averse, risk-neutral, and risk-seeking. We make a categorical independent variable that equals to 1 when a participant belongs to one of each risk-preference groups. We follow a similar procedure to examine the relationship in Experiment 1:

$$\begin{aligned} y_{i}\;=\rho _{0}+\rho _{1}{\text {probinf}}_{i}+\rho _{2}{\text {stat}}_{i}+\rho _{3} {\text {riskpref}}{}_{i\kappa }+\rho _{j}X_{i,j}^{'}+\xi _{i}, \end{aligned}$$

where $\xi _{i}\sim N\left( 0,\delta _{\xi }\right)$ and subscript $\kappa$ represents each agent’s risk preference: $\kappa =1$ for risk-averse, $\kappa =2$ for risk-neutral, and $\kappa =3$ for risk-seeking.

The following section deals with “unknown uncertainty” based on Knight’s claim.

### Maximization of uncertainty: unknown uncertainty

For maximizing uncertainty condition, we provide a future reward without any information: participants are provided a sealed envelope which contains the unknown reward. We expect that risk preference plays a key role in decision-making procedures under maximized uncertainty. We ask them the maximum and minimum expected amounts in the sealed envelope. They are also asked about the probability that the maximum amount of compensation predicted will be in the sealed envelope. Using their responses, the expected future reward of each participant in the sealed envelope is defined as follows:

4 $$\begin{aligned} {\mathbb {E}}\left[ {\text {Late}}_{{\text {envelop}}}\right] ={\mathbb {E}}\left[ p\right] \cdot R_{{\text {max}}}+(1-{\mathbb {E}}\left[ p\right] )\cdot R_{{\text {min}}}, \end{aligned}$$

where ${\mathbb {E}}\left[ p\right]$ is the expected probability that the maximum amount of compensation predicted will be in the envelope, $R_{{\text {max}}}$ is the maximum expected amount in the envelope, and $R_{{\text {min}}}$ is the minimum expected amount in the envelope. Using this expected value for far-future choices as far-future reward, we can calculate each discounted future utility discount parameter $\delta$ and present preference parameter $\beta$.

In Experiment 3-1, we provide no information about the far-future choice, and participants have to predict the reward amount in the sealed envelope. When they open the sealed envelope, each agent can capture the difference between her prediction and the actual rewards the agent receives. In this section, we first check how many discordant choices are made by participants under the maximization of uncertainty. According to ESM Table 5, 5.36% of 168 participants make discordant choices under maximized uncertainty. The number of discordant choices under maximization of uncertainty is the same as the number of discordant choices under uncertainty with the guaranteed far-future minimum. These results also support our argument that uncertainty allows participants to make fewer discordant choices and focus more on the decision-making procedures. However, maximizing uncertainty causes participants to be more confused in estimating future rewards than in other scenarios. We can see the confusion of the participants from their expected maximum far-future choices, the possibility of it, and their selection results. Some participants choose to get the sealed envelope in a year, even if their expected reward for the far-future is less than the amount of an alternative reward. In our experiments, an alternative reward for an immediate or a near-future reward is $1000. For example, one participant forecasts that the maximum and minimum amounts of reward in the sealed envelope is “$90,000” and “$100,” respectively. He also forecasts that the possibility of the maximum in the envelope is 0.01%. Then his expectation of reward in the envelope is “$999” which is smaller than “$1000,” but he chooses to wait a year for the reward in the sealed envelope. We call this kind of choice a self-contradictory choice. According to ESM Table 6 when predicting unknown future compensation compared to immediate compensation, it is found that 16.36% of participants with weak probabilistic inference make a self-contradictory choice. However, only a few of participants with strong probabilistic inference make a self-contradictory choice. As the delay in alternative payments increases, participants with weak probabilistic inference still make more self-contradictory choices than participants with strong probabilistic inference. We can explain these self-contradictory choices as a result of risk preference or misestimation for the unknown far-future reward. Participants who make self-contradictory choices might be a risk-seeker, or they may overestimate the far-future reward in the sealed envelope.

**Fig. 2:**
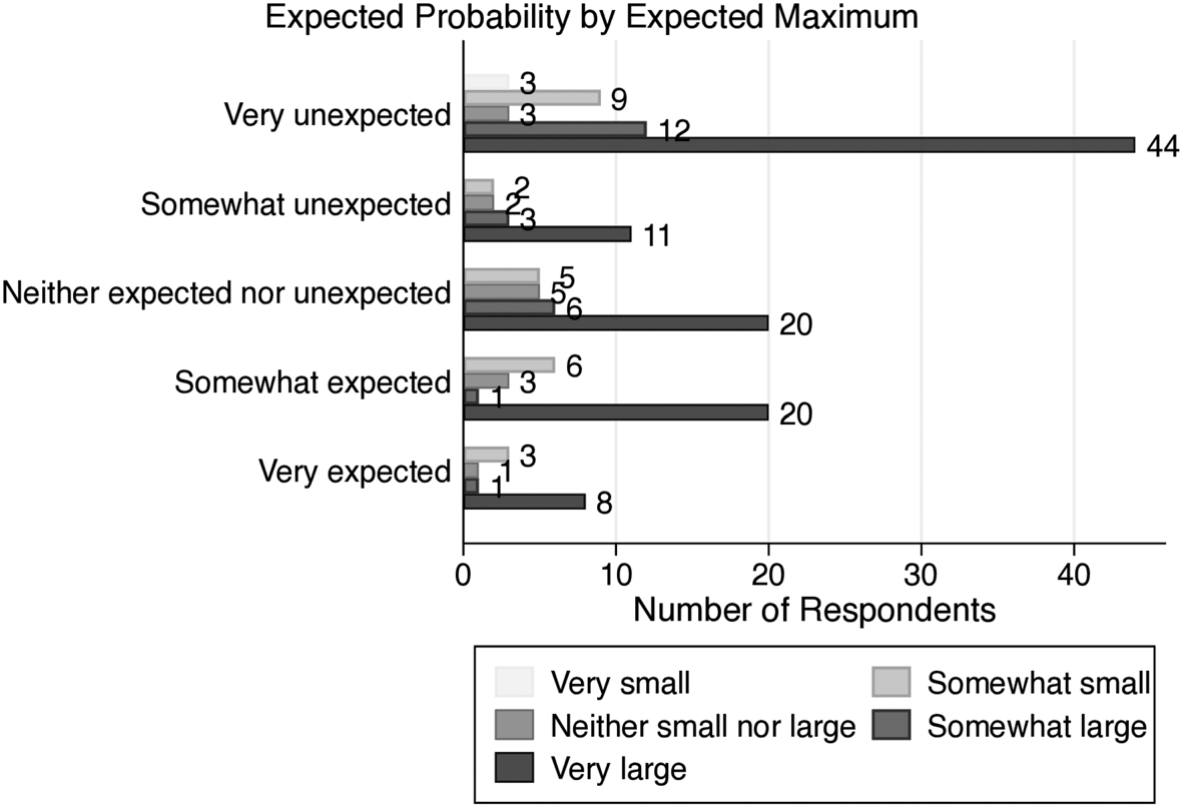
Number of respondents for forecasts under maximized uncertainty

Since the maximum compensation and the possibilities they forecast vary from person to person, we organize two categorical variables as follows: (1) 5 levels of expected maximum amount in the sealed envelope: very small $$[\$0,\$1000)$$, somewhat small $$[\$1000,\$2000)$$, neither small nor large $$[\$2000,\$4000)$$, somewhat large $$[\$4000,\$10,000]$$, and very large $$(\text {over }\$10,000)$$, (2) 5 levels of expected probability of subjective maximum in the sealed envelope: very unexpected [0, 0.2), somewhat unexpected [0.2, 0.4), neither unexpected nor expected [0.4, 0.6), somewhat expected [0.6, 0.8), and very expected [0.8, 1]. According to Fig. 2, 103 out of 168 participants predict that the maximum is over $10,000. Seventy-one participants predict that while their expectations are very low (less than 20%), nearly 65% of them are expecting more than the maximum amount $10,000.

**Fig. 3:**
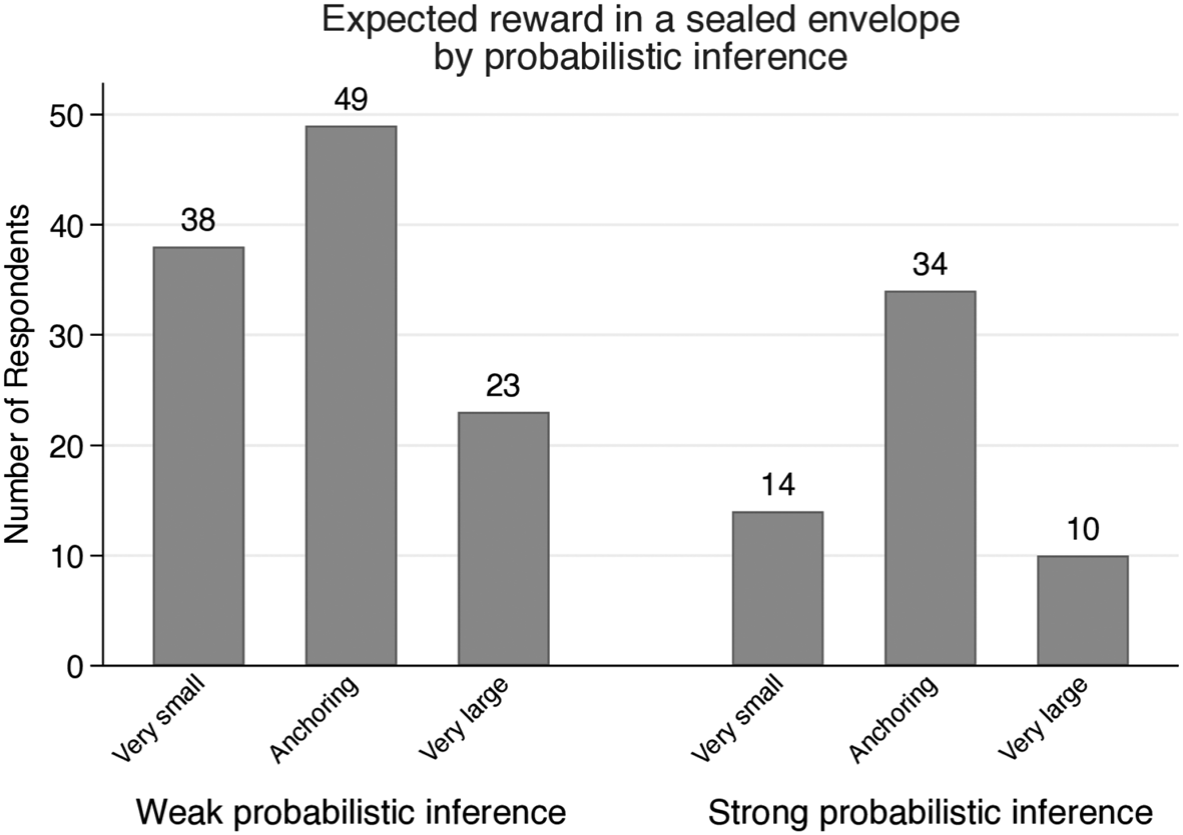
Distributions of expected future rewards by probabilistic inference

Figure 3 shows distributions of expected reward in the envelop by probabilistic inference level. Calculating the expected future rewards of individuals satisfying Eq. (4), nearly half (49.40%) of the participants expect the unknown future reward between $1000 and $10,000. Although we did not provide any information, their expectations do not deviate from the scale of compensation given in previous experiments. Here we can capture the anchoring effect from the previous experiments. The previous experiments produce anchoring bias, where participants may rely on pre-existing information, not given information. Even though the participants are told that each experiment is independent, they still act as if they were under the influence of the conditions of the previous experiments. The expected reward of participants with strong probabilistic inference is a bell-shaped distribution, and that of participants with weak probabilistic inference is a right-skewed distribution. Over thirty-four percent (34.55%) of participants with weak probabilistic inference expect very small amount (less than the alternative reward) for the future reward in the envelope. Nearly twenty-four percent (24.14%) of participants with strong probabilistic inference expect very small amount in the envelope.

The regression of this section measures the effect of the maximization of uncertainty and subjective forecasting. Maximized uncertainty exacerbate confusion in estimating the difference in ratios of future discount factors. Under maximized uncertainty, our data shows that most of respondents choose an immediate and small reward rather than an unknown far-future reward. This choice behavior most often generates the $\beta$ parameter that is close to 0, and eventually, the difference in marginal rate of substitutions, $\triangle {\text {MRS}}$, is always zero. Then this variable cannot be a proper dependent variable under maximized uncertainty, so we decide to examine the OLS regression only for the change in number of willingness to wait. As uncertainty is maximized, each participant has to forecast the far-future reward in the sealed envelope without any information. We make binary categorical independent variables: overestimate$_{i}$ and underestimate$_{i}$. They equal to one when a participant forecasts with overestimation or underestimation. The regression is as follows:

$$\begin{aligned} \begin{aligned} y_{i}&= \rho _{0} + \rho _{1}{\text {probinf}}_{i} + \rho _{2}{\text {stat}}_{i} + \rho _{3}{\text {riskpref}}{}_{i\kappa } + \rho _{4}{\text {overestimate}}_{i} \\&\quad + \rho _{5}{\text {underestimate}}_{i} + \rho _{j}X_{i,j}^{'} + \xi _{i}, \end{aligned} \end{aligned}$$

where $\xi _{i}\sim N\left( 0,\delta _{\xi }\right)$. We expect that risk preference does not affect the willingness to wait under maximized uncertainty. Unknown uncertainty exaggerates the fear of losses, and it hinders participants from choosing wait for a large reward.

### Informed uncertainty: Bayesian updating

Finally, we investigate the relationship between subjective probabilistic inference and future forecasting ability under the informed uncertainty. Our concerns are how much individuals understand and apply given information and whether they make positive or negative predictions when forecasting the future event. First, we check how many discordant and self-contradictory choices are made by participants under the informed uncertainty, respectively. ESM Table 5 explains that only 18 participants who have weak probabilistic inference make a discordant choice under this experiment. According to ESM Table 6, participants with strong probabilistic inference do not make a self-contradictory choice. As the delay in alternative early payments increases, some respondents might recognize that their own expectation is smaller than the alternative reward, and they switch their choices. Under the informed uncertainty, given information acts as the catalyst for decision-making procedures of participants with strong probabilistic inference.

**Fig. 4:**
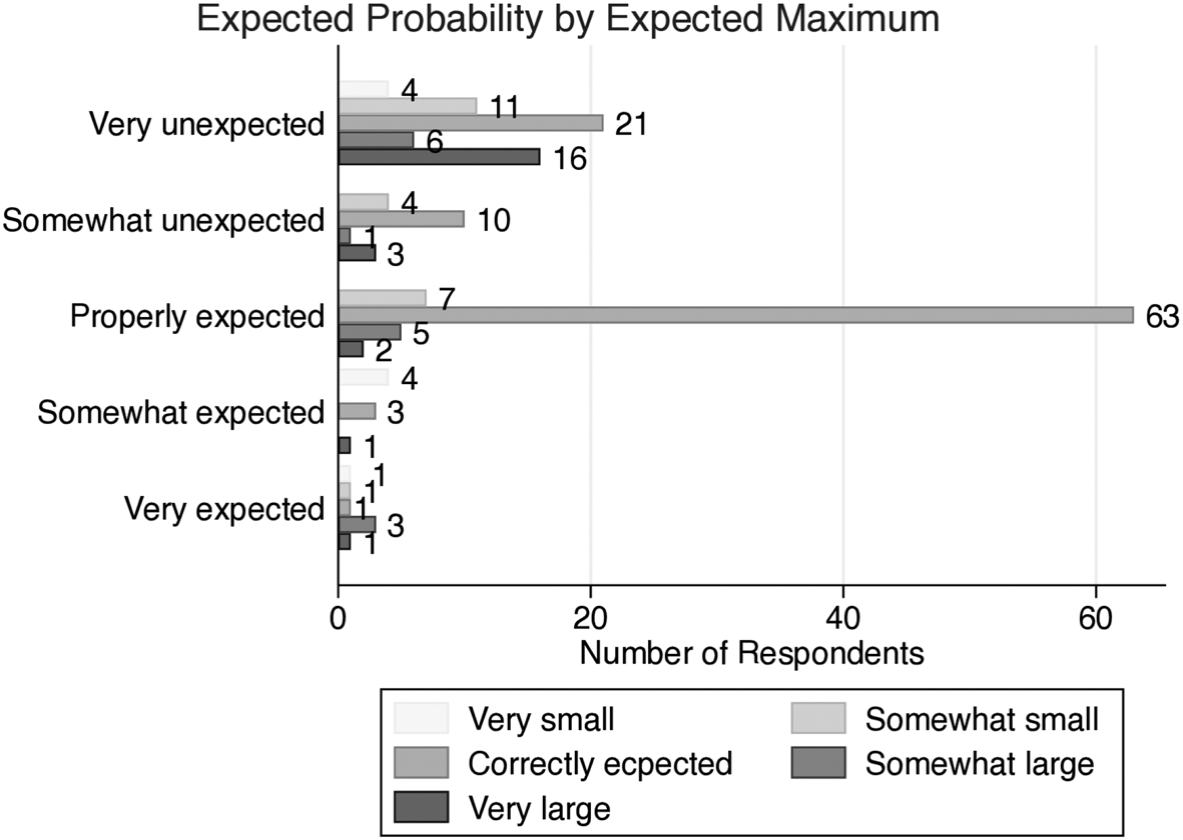
Percent of respondents for forecasts under informed uncertainty

Similar to “Maximization of uncertainty: unknown uncertainty”, we categorize individual’s expected maximum amount in the sealed envelope: very small, somewhat small, correctly forecast $$\left( \max r_{12}^{X}=\$5000\right)$$, somewhat large, and very large. Also, we use the other categorical variable: 5 levels of the expected probability for subjective maximum in the sealed envelope from “Maximization of uncertainty: unknown uncertainty”. We also use the same 5 levels of the expected probability. According to Fig. 4, 63 out of 168 participants accept the given information accurately. This shows that given information is applicable to their decision-making processes without ignoring it. More than 34 percent (34.52%) of participants do not expect the unknown reward in the sealed envelope to be greater than the alternative. Only 8.92% of participants expect the unknown reward is over $5000 which is over the given information.

**Fig. 5:**
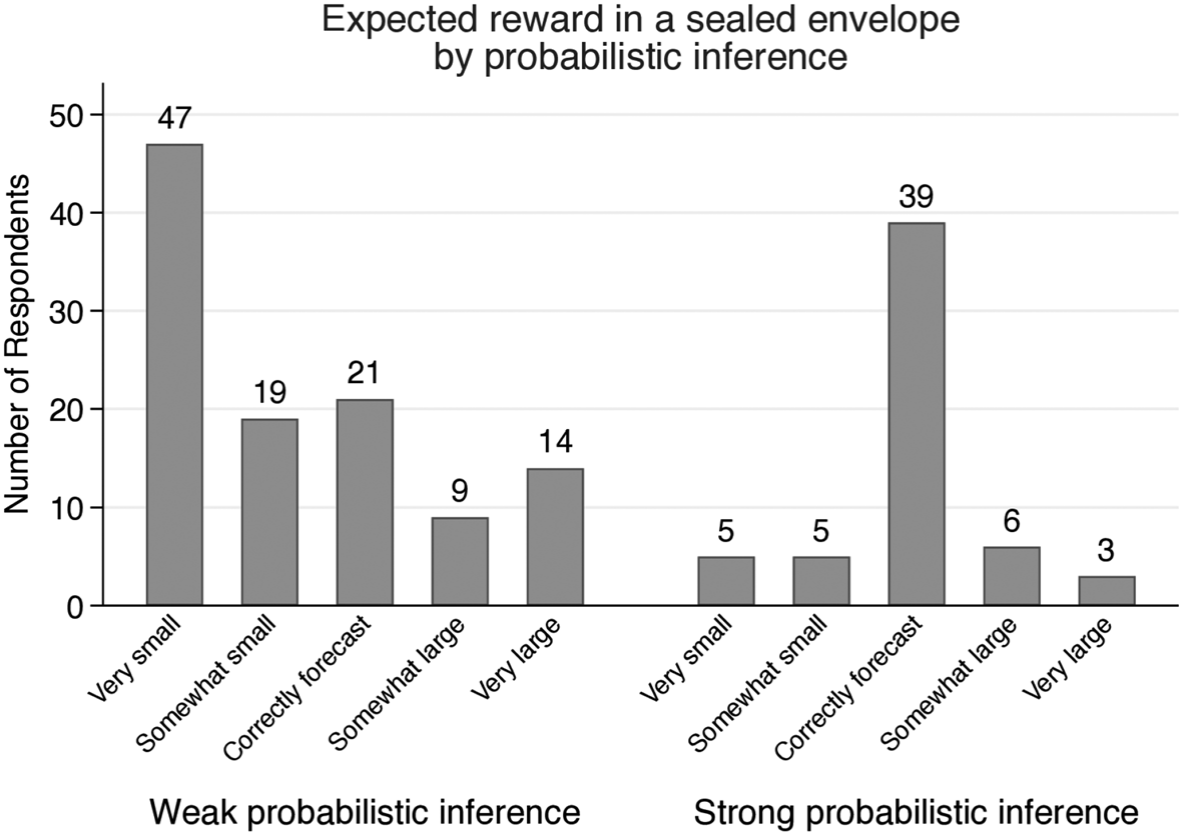
Expectation for far-future reward under informed uncertainty

We provide the distribution of expectations of the unknown reward by subjective probabilistic inference in Fig. 5. The distributions are a right-skewed and a bell-shaped for participants with weak- and with strong-probabilistic inference, respectively. Over nineteen percent (19.09%) of participants with weak probabilistic inference predict correctly, while 67.24% of participants with strong probabilistic inference predict correctly. Many (42.73%) of participants with weak probabilistic inference extremely underestimate (< $1000), and 12.73% of them extremely overestimate (> $10,000). We understand probabilistic inference can play an essential role in information acquisition. This subjective expectation could be the hidden own criteria in choices.

The last regression measures the effect of the informed uncertainty and subjective forecasting. We create a binary forecasting accuracy variable to distinguish between accepting given information correctly or not: $${\text {infoestimate}}_{i}$$. When a participant accepts and applies given information to predict the far-future reward in the envelope, $${\text {infoestimate}}_{i}$$ is $${\text {success}}$$ or $${\text {failure}}$$. For example, in our experiment, when we provide the information “$0; $0; $5000; $5000; $0; $5000” and participant *i* captures 50% of $5000 and 50% of $0 for the far-future reward: $$E\left[ {\text {Late}}\right] =0.5\times \$0+0.5\times \$5000=\$2500$$, then $${\text {infoestimate}}_{i}={\text {success}}$$. Otherwise, we define $${\text {infoestimate}}_{i}={\text {failure}}$$. Using this binary variable, we set up the OLS regression in the following manner:

$$\begin{aligned} \begin{aligned} y_{i}&= \rho _{0}+\rho _{1}{\text {infoestimate}}_{i}+\rho _{2}{\text {stat}}_{i} +\rho _{3}{\text {riskpref}}{}_{i\kappa }+\rho _{4}{\text {overestimate}}_{i}\,\\&\quad +\rho _{5}{\text {underestimate}}_{i}+\rho _{6}{\text {infoestimate}}_{i} +\rho _{j}X_{i,j}^{'}+\xi _{i},\, \end{aligned} \end{aligned}$$

where ${\text {cov}}\left( \varepsilon _{i},\xi _{i}\right) =0$. We examine choice differences that depend on information acquisition and forecasting. We expect that there is a positive relationship between subjective probabilistic inference and information application capability. Probabilistic inference affects information updating ability, and each participant sets up his own criteria for far-future choices. Each participant makes a decision by incorporating these own criteria into the decision-making process.

## Regression results

Many empirical studies suggest that a single concept of probability does not accurately capture people’s responses to uncertainty. Compared to the argument of Knight (1921) who made the distinction between cases of known probabilities and of unknown probabilities, this section outlines people’s intertemporal choices under known uncertainty and under unknown uncertainty, respectively. We also include robust regression results in each OLS regression result.

### Regression without uncertainty

**Fig. 6:**
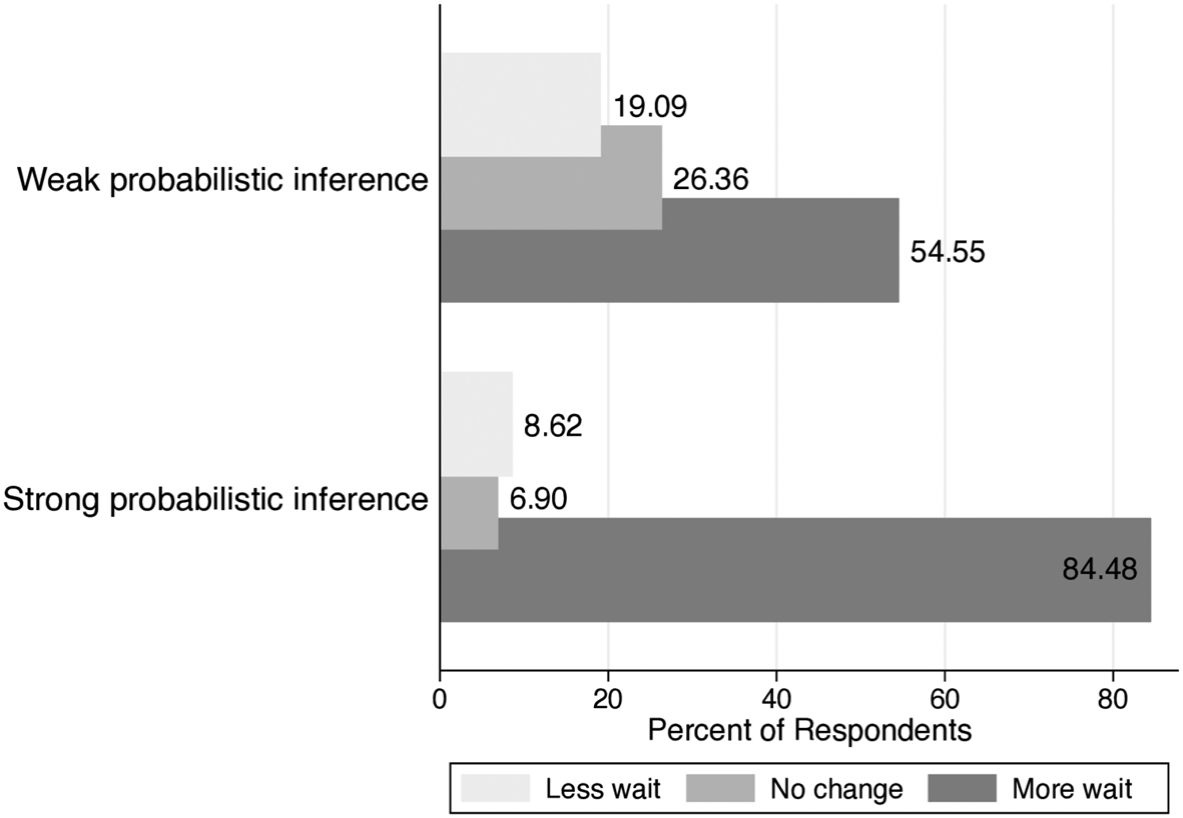
Change in willingness to wait by probabilistic inference without uncertainty

First, we examine the change in the number of willing to wait as delays increase, $\nabla WW$, which is satisfied in Eq. (2). The positive value of $\nabla {\text {WW}}$ represents that the agent is more willing to wait as delays increase. Figure 6 shows changes in willingness to wait by subjective probabilistic inference. Nearly eighty-five percent (84.48%) of participants with strong probabilistic inference decide to change their previous choices for greater compensation as the delay increases and wait another year. And 54.55% of participants with weak probabilistic inference decide to wait another year by reversing his or her choice not to wait. In addition to eliminating immediate rewards, probabilistic inference seems to have a strong effect on willingness to wait. This leads us to estimate the OLS regression of changes in willingness to wait, $\nabla {\text {WW}}$. We highlight outcomes that are significant at $p<0.10$ for a majority of estimates. ESM Table 7 shows that if probabilistic inference increases by 1%, the average ratio of willingness to wait for the far-future reward increases by near 60%. Once again, the result from OLS regression emphasizes that learning statistics, understanding the Bayes’ rule, and having good statistical knowledge lead to people waiting more as delay increases.

The willingness to wait also enables potential trade-offs to be analyzed using marginal rates of substitution. We provide the OLS results of the difference in marginal rate of substitutions in ESM Table 9. When immediate gains are delayed, a 1% increase in probabilistic inference and statistical knowledge, respectively, makes participants 30% and 89% weight for the utility of future rewards versus the alternative. But gender and majors are not significant in intertemporal choices without uncertainty.

### Regressions under known uncertainty

We propose that minimum guarantee information influences the attractiveness of willingness to wait for the far-future reward. Subjective risk preferences change with the degree of safety of given information. Compared to the outcomes of Experiment 1, if a participant changes his decision to wait a year less as delays increase, then the participant is risk-averse. We try to demystify this higher rate of willingness to wait under uncertainty. Theoretically, we expect that participants are more willing to wait without uncertainty. But our opposite outcomes require another explanation: the discordance of estimating future rewards. Even if uncertainty exists, the greater rewards of the future are attractive to many respondents. We confirm that they do not properly estimate far-future rewards, even though they are provided with the same expected values under certainty or uncertainty. We focus on synthetic effects of probabilistic inference and risk preferences in decision-making procedures under uncertainty.

#### Without the guaranteed minimum

**Fig. 7:**
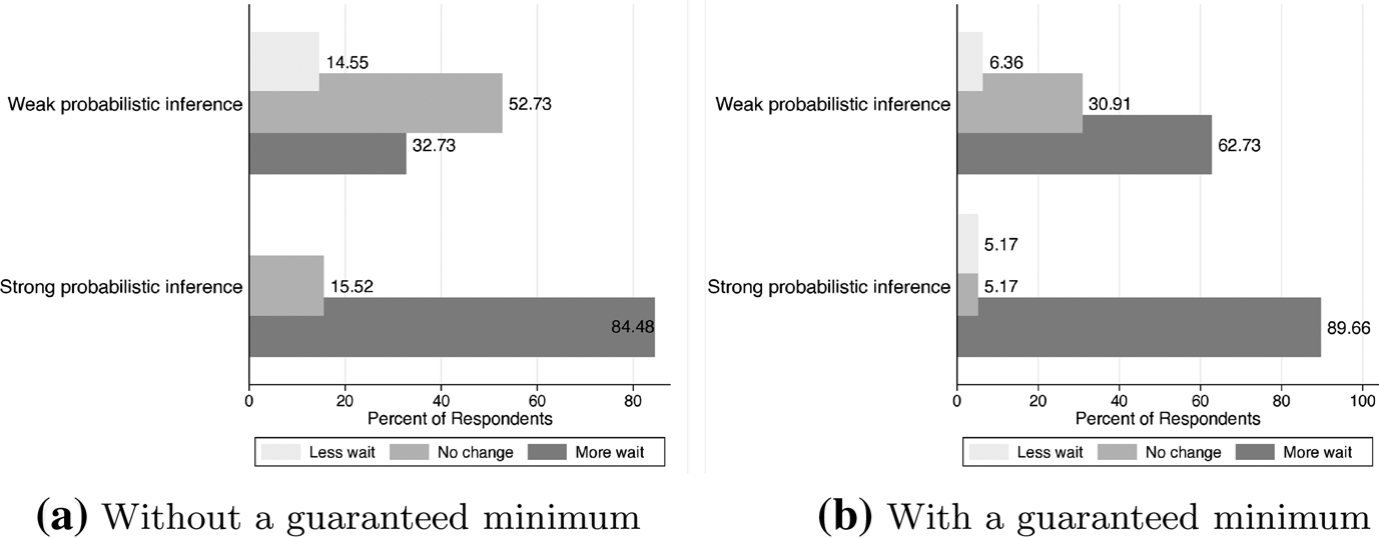
Changes in willingness to wait by probabilistic inference under known uncertainty

According to Fig. 7a, when the merits of immediate rewards have disappeared, over 52% of participants with lower level of subjective probabilistic inference adhere to the previous choices they made while only 15.52% of participants with stronger level of subjective probabilistic inference decide not to change their choices. Also, almost 15% of participants with low probabilistic inference decide not to wait another year by reversing his or her choice to wait, when they do not have any minimum information for the far-future reward. With lack of forecasting ability, they ignore the possibility that they may be profitable. At this point, we cannot exactly say that their choices are perfectly linked to their risk preferences. However, it goes without saying that the type of risk preference also plays an important role in the choice of time. We can explain that forecasting is the outcome of a combination of probabilistic inference and risk preference. According to ESM Table 8, even if there is a possibility of zero gains, men are 11.40% more willing to wait than women. We can explain that women are more risk-averse than men and this characteristic leads to their hesitation under uncertainty. In addition, we can explain that men tend to focus more on the amount of rewards they can receive than women. While probabilistic inference is not significant at any level compared to the results of the choice without uncertainty, STEM majors, learning statistics, and statistical knowledge make participants wait more for the far-future reward that does not provide guaranteed minimum.

In ESM Table 10, the OLS results of the difference in *MRS* provide explainable reasons of the above results: many participants do not make a big estimate of the utility of a far-future reward. Even if the minimum of future rewards is not guaranteed and immediate reward benefits are lost, STEM participants consider the utility of later but larger rewards 15–20% more important, and those who have learned statistics consider it 10% more important. Because of uncertainty, risk preferences are important role in decision making-procedures: while the risk-averse characteristic makes rewards in the far-future as less attractive as 46%, the risk-seeking characteristic makes it 73% more attractive. Other factors, however, do not have much impact. Compared to the absence of uncertainty, under uncertainty where minimum future reward is not guaranteed, individual probabilistic inference factor does not play a major role in making future reward attractive. The possibility of greater losses evolves risks in decision-making procedures and hinders the exercise of subjective probabilistic inference.

#### With the guaranteed minimum

This section can explain the effect of a guaranteed information under uncertainty on decision-making procedures. The minimum guarantee criteria can make less fear of losses. First, Fig. 7b shows that the guaranteed minimum payoff affects more strongly the choices of participants with weak probabilistic inference than those with strong inference. They are more willing to wait than before. Here, nearly 62% of participants with lower level of subjective probabilistic inference also decide to reverse their previous choices and wait more periods. We also estimate the OLS regression of changes in willingness to wait, $\nabla {\text {WW}}$, under uncertainty with the guaranteed minimum information. From ESM Table 8, when participants are provided the minimum information for the far-future reward under uncertainty, the subjective probabilistic inference affects positively the willingness to wait. This factor might help to capture given information for positive forecasting. Lossless relief under uncertainty makes risk-seeking participants more willing to wait and risk-averse participants less willing to wait. But while the risk-seeking participants seem less willing to wait than in previous experiments (from 73 to 43%), this is the result of their choices of future rewards, whether immediate reward is possible or not. Other independent variables including learning statistics and levels of statistical knowledge are not significant at any level.

From the OLS results of the difference in MRS in ESM Table 10, we capture that when participants get the non-zero guaranteed minimum for far-future choices even if there still exists uncertainty, one percent increase in probabilistic inference, learned statistics, and statistical knowledge attainment leads to 57.83%, 20.75%, and 26.46% higher the difference in MRS, respectively. With the minimum safeguard for losses, these abilities positively affect the utility of long-term rewards. We focus on that higher probabilistic inference less discount the future utility if the minimum reward is guaranteed under uncertainty. In addition, thanks to the guaranteed minimum gain, men value future rewards 28% more than women. In terms of risk preference, risk-averse is still significant at a 1% level, but risk-seeking is not. Risk-averse participants estimate nearly thirty percent (29.60%) less utility for far-future rewards when there is a guaranteed minimum, which is a marked reduction: when there is no minimum guarantee, they estimate 97.84% less utility. Due to the positive effect of a guaranteed minimum under uncertainty, risk-seeking characteristic does not significantly affect differences in MRSs because they always place more value on future rewards when alternatives are immediate or not. Besides that, STEM majors also have a positive impact. Uncertainty dampens long-term investor sentiment and makes participants prefer immediate payoffs. By presenting a minimum safeguard against losses due to uncertainty, the expected profits are the same mathematically as before, but participants focus on the maximum profits they can receive, leading them to seek future rewards. Even risk-averse participants are more willing to wait a year if they reduce the risk burden. This safeguard leads to reversal of time preference when the charm of immediate benefits is removed. Less fear of losses encourages many participants to wait a year for the bigger reward.

### Regressions under maximized uncertainty

“Regressions under maximized uncertainty” contributes to the argument of Knight (1921). Maximized uncertainty induces pre-choice situations in which participants must predict unknown future rewards. Participants should predict the future rewards in the envelope compared to a given alternative reward and choose between the alternative reward and their own predictions, which may be overestimates or underestimates and may be anchored to the rewards amounts of other experiments. In our experiment, we define that an overestimation is when the prediction of the amount of reward in the envelope is over $10,000, and an underestimation is when the prediction is less than $1000. We provide information on the subjective estimation variable in ESM Table 11. Fifty-one out of 168 respondents underestimate and 28 respondents overestimate the amount in the sealed envelope. There are slightly more men than women who underestimate unknown rewards in the value expectation distribution. Nearly fifty-three percent of respondents predict the value of their far-future choice within the dollar amount of the reward given in the previous experiments. We assume that the choice conditions of previous experiments unconsciously affect some participants’ forecasting mechanisms: anchoring effect. Previous experiments leave the afterimage in an individual’s unconscious and interfere with the next choices. The weaker the probabilistic inference of a participant, the more overvalued the rewards in the sealed envelope. In addition, many participants forecast losses in sealed envelopes, even if the appeal of immediate compensation disappears, they want to pursue solid gains. We can confirm that fear of losses from maximized uncertainty plays a central role in whether they are willing to wait.

Under maximized uncertainty, our data supports the result of Anderson and Stafford (2009) who explain that increased risk reduces individual’s patience. We provide the OLS regressions of willingness to wait in ESM Table 13. From this OLS regression result, From this OLS regression result, other than the degree of individual estimates and the probabilistic inference variables, no variables induce significant results in the willingness to wait. Underestimation of the unknown reward is significant at a 5% level and we can explain that unknown uncertainty hinders participants from making optimistic predictions about the reward. Probabilistic inference is also significant at decision of waiting. 1% increase in probabilistic inference induces 29.57% of increase in willingness to wait when there are no attractiveness of immediate gains. As uncertainty maximizes, participants’ risk preferences do not significantly affect their choices. Because of the fear of losses, many participants give up their options that may be heavily compensated. Even risk-seeking participants do not want to take a risk choosing larger rewards under unknown uncertainty. This result shows that uncertainty associated with risk preferences affects each individual’s choice, and also uncertainty itself affects choices. Unknown uncertainty neutralizes influence of each individual’s risk preference. And it induces things participants neither aware of nor understand, and it negatively affects willingness to wait.

### Regression under the informed uncertainty

In this section, we focus on participants’ information acquisition capability. Others’ choice results may be underlying probability information for personal predictions. We start with presenting information on the respondents’ forecasting accuracy in ESM Table 12. Over sixty-one percent (61.90%) respondents have failed to provide accurate forecasting information. The distribution of predictive accuracy between men and women is similar. STEM major has a positive effect on making accurate predictions. In addition, the result shows that good statistical knowledge, learning statistics, and strong probabilistic inference help individuals to understand and accept given information.

In the last regression, we use the variable ${\text {infoestimate}}_{i}$ as our new independent variable which represents accuracy of forecasts. ESM Table 13 show that learning statistics, probabilistic inference, and accuracy of forecasting are significant at a 1% level. In the aspect of information acquisition capability, participants who make accurate predictions along with given information will be more willing to wait another periods. They recognize the probability of making a profit, and try to bet for the bigger rewards. Also, when the charm of immediate gains disappear, 1% each increase in the levels of probabilistic inference and statistical knowledge make 37.57% and 32.95% increases in willingness to wait, respectively. We interpret that participants who attain strong probabilistic inference can predict more accurately the unknown reward than others. STEM majors have positive effect on willingness to wait, but there is no gender effect. Risk-seeing is not significant but risk-averse positively affect willingness to wait. Informed information makes risk-averse participants choose to wait more when there is no immediate benefits. Many of risk-seeking participants always choose to wait for the larger reward whether the alternative is immediate or not. This fact makes that risk-seeking characteristic is not significant in difference in willingness to wait.

This OLS regression emphasizes that when participants are given information that they can guess even if there still exists uncertainty, learning statistics, understanding the Bayes’ rule, and having good statistical knowledge positively affect willingness to wait as the immediate reward option is gone. We capture that the capability of information acquisition for forecasting more affect the willingness to wait. We can explain that the capability of information acquisition is related to forecast ability and this capacity can be a hidden subjective criterion in intertemporal choices. Participants who have strong probabilistic inference accept and apply given information accurately, and their willingness to wait increases as the alternative payment is postponed. Unlike the results in totally unknown uncertainty, even risk-averse characteristic shows an optimistic will due to the given information and postpone of immediate reward.

## Conclusion

Important decisions made at many points in an individual’s life include trade-offs over payoffs at alternative points in time. This paper aims to explore important determinants of decision-making procedures in intertemporal choices under uncertainty. Our findings are experimental manifestations of Knight’s argument (1921): difference between known uncertainty and unknown uncertainty. To address the role of uncertainty in decision-making procedures, we conduct a unique field experiment, and we examine the effects of subjective probabilistic inference on intertemporal choices under uncertainty. In the midst of uncertainty, we find that not only risk-seekers but also participants who have strong probabilistic inference are more likely to be willing to wait for a large reward as the attractiveness of immediate gains diminishes, especially when information is given that can be inferred.

Two implications can be derived from this finding. First, the capability of information acquisition may trigger an increase or decrease of willingness to wait. Here, this ability results in forecasting utilities of future events. Probabilistic inference relates to the existence of available information in choice criteria. The flexibility of thinking are essential in finding more accurate answers. We capture that agents with strong probabilistic inference accept and apply more accurately given information than agents with weak probabilistic inference. We find that although large compensation is not guaranteed, a loss-free guarantee option allows an individual’s risk preference to play a significant role in the decision-making process. Minimum guarantee information influences the attractiveness of willingness to wait for the far-future reward. When people recognize uncertainty but also understand the existence of a guaranteed non-zero minimum gain in choice criteria, the subjective probabilistic inference has a positive impact on the willingness to wait as delays increase, otherwise it has little effect. In addition, when information is given that can be inferred under unknown uncertainty, we find that subjective probabilistic inference has a great effect on decision-making procedures. This finding is consistent with the result of Benjamin et al. (2013). Probabilistic inference affects constructing subjective criteria which were predicted using given information, and then risk preferences combine with these subjective criteria to affect time preferences. In addition, discordant or self-contradictory choices are the outcomes of misunderstanding, overestimation, or underestimation of given information. This finding can be an evidence for the result of Steiner and Stewart (2016). Other variables including gender, statistical knowledge, and learned statistics can also explain changes in intertemporal choices as the initial alternative payment is postponed.

Second, our results reflect the impact of the fear of losses on choices under unknown uncertainty. Many studies argue that uncertainty creates a fear of loss that it results in each choice as an expression of individual risk preferences. However, our experiment shows that fear of loss can offset the influence of risk preference, which makes many participants hesitate to wait, including risk-seekers. The higher possibility of no gains for far-future rewards makes people hesitate to wait. Risk preference does not play an essential role in decision-making procedures under unknown uncertainty. This finding can support the researches of Fréchette et al. (2017) and Golman and Loewenstein (2018). However, subjective probabilistic inference has effect on willingness to wait under unknown uncertainty. Self-acquired information by forecasts serves as a catalyst in the decision-making process. We can explain that people’s own subjective forecasting for the unknown reward turns to be a hidden criterion in intertemporal choices. Fear of losses due to increased uncertainty turns into an obstacle to the information acquisition process, and especially agents with low probabilistic inference tend to overestimate or underestimate future rewards.

In all, the results presented here support the idea that a deeper understanding of cognitive bias can be achieved through the application of individual-difference research, paying more attention to the subjective probabilistic inference and different types of risk preferences. Our study illustrates the relevance of implementing field experiments to provide further insights into prospect theory. Also, in addition to time preferences and risk preferences, we explore the role of information in decision-making procedures. Although much work remains to be done, current research makes a strong case for the value of forecasting in intertemporal choices. Subjective probabilistic inference plays a central role in many everyday cases of forecasting, and as the result of forecasting exerts substantive constraints of restriction or stimulus on cognitive processes or decision-making procedures. A key goal of this study is to measure the practical empirical importance of these different theoretical mechanisms. Ultimately, we do not know the correct answer to the methodological research design, but we can propose this study as an example for future research. Some limitations of the study must be considered when interpreting the experimental results. First, the task in our experiments are simpler than many actual decision-making scenarios in which the decision environment is much more complex. Second, the computation of the base rate fallacy, as well as the measurement of statistical knowledge, might be controversial. Next, the study was conducted on a relatively limited sample size, which may have reduced the statistical power of our results. We expect to complete an experiment with large sample size to understand why individuals have behaved inconsistently in intertemporal choices. Finally, the majority of agents are college students from the same age group, it is possible that non-significant results can occur from the fact. Nevertheless, we believe this technique is applicable to other domains.

## Supplementary Information

Below is the link to the electronic supplementary material.

Supplementary file1 (DO 38 KB)

Supplementary file2 (CSV 95 KB)

Supplementary file3 (PDF 213 KB)
