# Supplementary material for: Too much or too little information: how unknown uncertainty fuels time inconsistency
Source: SN Bus Econ. 2022 Jan 19;2(2):17. doi: 10.1007/s43546-021-00189-9 (PMC8767536; doi:10.1007/s43546-021-00189-9)
Supplement: Supplementary file 3 — Supplementary file3 (PDF 213 KB) [file 43546_2021_189_MOESM3_ESM.pdf]

Appendix

Tables

Demographic characteristics		Respondents #	Respondents %
Gender	Male	82	48.81
	Female	86	51.19
Age	18 - 22	133	79.17
	22 - 39	17	10.12
	30 - 39	12	7.14
	40 - 59	6	3.57
Major	Arts-related	16	9.52
	Business-related	49	29.17
	Math & Science related	39	23.21
	Engineering & Technology	23	13.69
	Literature, Language, & Social Science	20	11.90
	Others	21	12.50
Statistics	Yes	88	52.38
	No	80	47.62
Financial items	Saving Account	147	87.50
	CDs	47	27.98
	Auto Loan	36	21.43
	Student Loan	78	46.43
Statistical Knowledge	Good	28	16.67
	Normal	77	45.83
	Poor	63	37.50

*Total respondents # = 168

Table 1: Demographic characteristics of respondents

		Statistical Knowledge				Total #
		Poor	Normal	Good	Mean (SD)	
**Statistical Knowledge**	Poor	63			0.1905 (0.1073)	63
	Normal		77		0.5 (0)	77
	Good			28	0.8393 (0.1220)	28
**Gender**	Male	29	38	15	0.4482 (0.2381)	82
	Female	34	39	13	0.4331 (0.2468)	86
**Majors**	Mathematics/Science	11	18	10	0.5128 (0.2625)	39
	Engineering/Technology	3	10	10	0.5870 (0.1937)	23
	Business	24	21	4	0.3724 (0.2345)	49
	Literature/Social	11	6	3	0.3875 (0.2864)	20
	Arts	8	8	0	0.3437 (0.1797)	16
	Others	6	14	1	0.4285 (0.1609)	21
**Financial Items**	Savings	53	68	26	0.4490 (0.2411)	147
	No savings	10	9	2	0.3810 (0.2452)	21
	Auto-loan	11	18	7	0.4931 (0.2688)	36
	No auto-loan	52	59	21	0.4261 (0.2347)	132
**Statistics**	Yes	28	40	21	0.4803 (0.2534)	89
	No	35	37	7	0.3955 (0.2215)	79
Mean (SD)		0.1905 (0.1079)	0.5 (0)	0.8393 (0.1220)	0.4425 (0.2420)	
Total #		63	77	28		168

Table 2: Number of respondents on statistical knowledge by variables

		Subjective Probabilistic Inference			Total #
		Strong	Weak	Mean (SD)	
**Statistical Knowledge**	Poor	11	52	0.2471 (0.2466)	63
	Normal	24	53	0.3616 (0.2795)	77
	Good	23	5	0.6812 (0.2356)	28
**Gender**	Male	24	58	0.3188 (0.2807)	82
	Female	34	52	0.4226 (0.3075)	86
**Majors**	Mathematics/Science	29	10	0.6402 (0.2049)	39
	Engineering/Technology	10	3	0.6686 (0.1684)	23
	Business	6	43	0.2430 (0.2427)	49
	Literature/Social	1	19	0.2215 (0.2098)	20
	Arts	0	16	0.1214 (0.1671)	16
	Others	2	19	0.1838 (0.1904)	21
**Financial Items**	Savings	56	91	0.3921 (0.2988)	147
	No savings	2	19	0.2310 (0.2609)	21
	Auto-loan	9	27	0.3342 (0.3077)	36
	No auto-loan	49	83	0.3822 (0.2962)	132
**Statistics**	Yes	43	46	0.4884 (0.2910)	89
	No	15	64	0.2435 (0.2548)	79
Mean (SD)		0.6925 (0.1699)	0.2029 (0.1945)	0.3719 (0.2984)	
Total #		58	110		168

Table 3: Number of respondents on subjective probabilistic inference by variables

Early reward: $1,000

Example	Rational case		Discordant case	
Late reward	today vs. in a year	in 6 months vs. in a year	today vs. in a year	in 6 months vs. in a year
$1,020	Refuse	Refuse	Refuse	Refuse
$1,100	Refuse	Accept	Refuse	Refuse
$1,500	Accept	Accept	Accept	Refuse
$2,000	Accept	Accept	Accept	Refuse
$10,000	Accept	Accept	Accept	Refuse

Table 4: Example of rational and discordant choice

Type of probabilistic inference	Strong		Weak	
Type of choices	Rational	Discordant	Rational	Discordant
Without uncertainty	53<br>(0.6936)	5<br>(0.6806)	89<br>(0.2277)	21<br>(0.0980)
Under uncertainty<br>No guaranteed minimum	58<br>(0.6925)	0<br>–	94<br>(0.2213)	16<br>(0.0949)
Under uncertainty<br>With guaranteed minimum	56<br>(0.6922)	2<br>(0.7011)	103<br>(0.2113)	7<br>(0.0791)
Maximized uncertainty	57<br>(0.6955)	1<br>(0.5215)	102<br>(0.2098)	8<br>(0.1151)
Informed uncertainty	58<br>(0.6925)	0<br>–	92<br>(0.2975)	18<br>(0.1701)
Total	58<br>(0.6925)		110<br>(0.2029)	

# of respondents (Mean probabilistic inference)

Table 5: Probabilistic inference by rational and discordant choices

Uncertainty	Choices	Strong PI		Total	Weak PI		Total
		Self-C	Normal		Self-C	Normal	
Maximized	Today vs. 1 year	1	57	58	18	92	110
	6 months vs. 1 year	2	56		16	94	
Informed	Today vs. 1 year	0	58		16	94	
	6 months vs. 1 year	0	58		7	103	

# of respondents,PI = probabilistic inference, Self-C = self-contradictory

Table 6: Self-contradictory choices by probabilistic inference

Ordinary Least Squares Regression (OLS) model for ∇WW.

	Without Uncertainty			
	Coefficient	S.E.	t	p-valued
Gender (Male)	- 0.0670	0.18 (0.10)	- 0.57	0.570 (0.486)
Arts-related	- 0.1672	0.25 (0.28)	- 0.66	0.512 (0.553)
Business-related	- 0.2643	0.20 (0.25)	- 1.33	0.184 (0.292)
Math & Science related	- 0.2278	0.24 (0.23)	- 0.96	0.339 (0.362)
Engineering & Technology	- 0.3263	0.26 (0.27)	- 1.25	0.214 (0.230)
Literature & Social Science	- 0.1670	0.23 (0.26)	- 0.72	0.470 (0.516)
Statistics	0.2675	0.13 (0.09)	2.09	0.038** (0.007)
Saving Accounts	0.0169	0.18 (0.13)	0.09	0.925 (0.879)
Auto Loans	- 0.0143	0.15 (0.12)	- 0.10	0.922 (0.885)
Residency (Urban)	0.0731	0.12 (0.12)	0.62	0.533 (0.498)
Probabilistic inference	0.5999	0.32 (0.27)	1.89	0.061* (0.028)
Statistical Knowledge	1.6376	0.21 (0.33)	5.99	0.000*** (0.000)
Intercept	- 0.4293	0.27 (0.32)	- 1.61	0.109 (0.179)

This is for the cases where subjects receive clear information without uncertainty..

The table presents an OLS regression for the changes in willingness to wait in percentage points.

Robust standard errors and p-value in parentheses. * $p < 0.1$, ** $p < 0.05$, *** $p < 0.01$.

Table 7: OLS regression for the changes in willingness to wait without uncertainty

Ordinary Least Squares Regression (OLS) model for ∇WW.

	Under Known Uncertainty							
	Without a guaranteed minimum				With a guaranteed minimum			
	Coefficient	S.E.	t	p-valued	Coefficient	S.E.	t	p-valued
Gender (Male)	0.1140	0.05 (0.04)	2.54	0.012** (0.005)	- 0.3489	0.11 (0.11)	- 3.15	0.002*** (0.002)
Arts-related	- 0.0172	0.09 (0.05)	- 0.18	0.855 (0.714)	- 0.0145	0.19 (0.14)	- 0.08	0.940 (0.918)
Business-related	0.0420	0.07 (0.05)	0.57	0.567 (0.410)	- 0.0041	0.15 (0.10)	- 0.03	0.978 (0.966)
Math & Science related	0.2678	0.09 (0.07)	3.04	0.003*** (0.000)	0.1127	0.18 (0.13)	0.64	0.526 (0.382)
Engineering & Technology	0.3172	0.10 (0.12)	3.24	0.001*** (0.007)	- 0.1293	0.20 (0.15)	- 0.66	0.509 (0.386)
Literature & Social Science	- 0.0306	0.09 (0.05)	-0.36	0.722 (0.502)	0.0253	0.17 (0.12)	0.15	0.884 (0.826)
Statistics	0.1835	0.05 (0.04)	3.75	0.000*** (0.000)	- 0.1527	0.12 (0.09)	- 1.28	0.202 (0.075)
Saving Accounts	0.0121	0.07 (0.06)	0.18	0.854 (0.835)	0.0796	0.13 (0.08)	0.61	0.546 (0.336)
Auto Loans	0.0064	0.06 (0.06)	0.12	0.906 (0.915)	0.0656	0.11 (0.10)	0.61	0.540 (0.503)
Residency (Urban)	0.0178	0.04 (0.04)	0.41	0.684 (0.666)	0.1045	0.09 (0.08)	1.21	0.227 (0.213)
Probabilistic inference	- 0.0918	0.12 (0.13)	- 0.77	0.444 (0.483)	0.5403	0.25 (0.22)	2.16	0.032** (0.014)
Statistical Knowledge	0.2278	0.10 (0.11)	2.26	0.025** (0.043)	0.2608	0.20 (0.19)	1.29	0.198 (0.179)
Risk-Averse	- 0.4578	0.089 (0.04)	- 5.80	0.000*** (0.000)	- 0.4657	0.12 (0.09)	- 3.77	0.000*** (0.000)
Risk- Seeker	0.7277	0.06 (0.08)	12.13	0.000*** (0.000)	0.4309	0.13 (0.13)	3.22	0.002*** (0.001)
Intercept	- 0.1401	0.10 (0.08)	- 1.40	0.162 (0.076)	0.2476	0.21 (0.15)	1.16	0.249 (0.108)

These are for the cases where subjects receive clear information under uncertainty: including the guaranteed minimum or not.

The table presents OLS regressions for the changes in willingness to wait in percentage points.

Robust standard errors and p-value in parentheses. * $p < 0.1$, ** $p < 0.05$, *** $p < 0.01$.

Table 8: OLS regression for the changes in willingness to wait under known uncertainty

Ordinary Least Squares Regression (OLS) model for $\triangle MRS$.

	Without Uncertainty			
	Coefficient	S.E.	t	p-valued
Gender (Male)	- 0.0167	0.07 (0.06)	- 0.25	0.801 (0.791)
Arts-related	0.0135	0.14 (0.17)	0.09	0.925 0.936)
Business-related	- 0.0253	0.11 (0.13)	- 0.23	0.820 (0.841)
Math & Science related	- 0.1324	0.13 (0.13)	- 0.99	0.322 (0.317)
Engineering & Technology	- 0.1179	0.15 (0.14)	- 0.80	0.422 (0.385)
Literature & Social Science	0.0149	0.13 (0.13)	0.12	0.908 (0.912)
Statistics	0.2181	0.07 (0.07)	3.05	0.003*** (0.003)
Saving Accounts	0.0522	0.10 (0.11)	0.52	0.602 (0.637)
Auto Loans	0.0235	0.10 (0.07)	0.52	0.602 (0.738)
Residency (Urban)	0.1107	0.07 (0.07)	1.69	0.094* (0.101)
Probabilistic inference	0.3023	0.18 (0.16)	1.70	0.092* (0.058)
Statistical Knowledge	0.8943	0.15 (0.16)	5.84	0.000*** (0.000)
Intercept	- 0.6423	0.15 (0.19)	- 4.30	0.000*** (0.001)

This is for the cases where subjects receive clear information without uncertainty..

The table presents an OLS regression for the differences in marginal rate of substitutes in percentage points.

Robust standard errors and p-value in parentheses. * $p < 0.1$, ** $p < 0.05$, *** $p < 0.01$.

Table 9: OLS regression for the differences in MRS without uncertainty

Ordinary Least Squares Regression (OLS) model for $\triangle MRS$.

	Under Known Uncertainty							
	Without a guaranteed minimum				With a guaranteed minimum			
	Coefficient	S.E.	t	p-valued	Coefficient	S.E.	t	p-valued
Gender (Male)	0.0660	0.03 (0.03)	2.19	0.030** (0.042)	0.2773	0.06 (0.06)	4.97	0.000*** (0.001)
Arts-related	0.0051	0.06 (0.04)	0.08	0.936 (0.906)	- 0.0129	0.10 (0.13)	- 0.13	0.894 (0.920)
Business-related	0.0665	0.05 (0.03)	1.35	0.178 (0.059)	0.0524	0.07 (0.09)	0.71	0.477 (0.570)
Math & Science related	0.1455	0.06 (0.04)	2.46	0.015** (0.001)	0.1840	0.09 (0.09)	2.06	0.041** (0.039)
Engineering & Technology	0.1984	0.07 (0.06)	3.02	0.003*** (0.002)	0.1977	0.10 (0.09)	2.01	0.047** (0.032)
Literature & Social Science	0.279	0.06 (0.04)	0.48	0.630 (0.436)	0.0647	0.09 (0.10)	0.74	0.461 (0.521)
Statistics	0.1062	0.03 (0.03)	3.23	0.002*** (0.001)	0.2075	0.06 (0.06)	3.46	0.001*** (0.001)
Saving Accounts	0.0335	0.04 (0.06)	0.76	0.451 (0.607)	- 0.0068	0.07 (0.10)	- 0.10	0.918 (0.946)
Auto Loans	- 0.0380	0.04 (0.05)	- 1.04	0.300 (0.456)	0.0480	0.05 (0.06)	0.89	0.374 (0.397)
Residency (Urban)	- 0.0267	0.03 (0.03)	- 0.91	0.364 (0.387)	- 0.0424	0.04 (0.04)	- 0.98	0.331 (0.341)
Probabilistic inference	- 0.0674	0.08 (0.07)	- 0.84	0.403 (0.363)	0.5783	0.13 (0.12)	4.59	0.000*** (0.000)
Statistical Knowledge	0.0706	0.07 (0.06)	1.04	0.299 (0.269)	0.2646	0.10 (0.09)	2.60	0.010*** (0.004)
Risk-Averse	- 0.9784	0.05 (0.05)	- 18.46	0.000*** (0.000)	- 0.2960	0.06 (0.07)	- 4.76	0.000*** (0.000)
Risk- Seeker	0.2493	0.04 (0.04)	6.19	0.000*** (0.000)	- 0.1035	0.07 (0.06)	- 1.53	0.127 (0.109)
Intercept	- 0.0716	0.07 (0.08)	- 1.07	0.287 (0.376)	- 0.3458	0.11 (0.17)	- 3.20	0.002*** (0.044)

These are for the cases where subjects receive clear information under uncertainty: including the guaranteed minimum or not.

The table presents OLS regressions for the differences in marginal rate of substitutions in percentage points.

Robust standard errors and p-value in parentheses. * $p < 0.1$, ** $p < 0.05$, *** $p < 0.01$.

Table 10: OLS regression for the differences in MRS under known uncertainty

(%) of respondents

Prediction		Underestimation	Normal	Overestimation	Total
Gender	Male	27 (32.93)	41 (50.00)	14 (17.07)	82
	Female	24 (27.91)	48 (55.81)	14 (16.28)	86
Major	Arts-related	3 (18.75)	10 (62.50)	3 (18.75)	16
	Business-related	20 (40.82)	18 (36.73)	11 (22.45)	49
	Math & Science	8 (20.51)	26 (66.67)	5 (12.82)	39
	Engineering & Tech	8 (20.51)	13 (56.52)	4 (17.39)	23
	Literature & Social Sci	5 (25.00)	11 (55.00)	4 (20.00)	20
	Others	9 (42.86)	11 (52.38)	1 (4.76)	21
Statistics	Yes	25 (28.09)	48 (53.93)	16 (17.98)	89
	No	26 (32.91)	41 (51.90)	12 (15.19)	79
PI	Weak	37 (33.64)	54 (49.09)	19 (17.27)	110
	Strong	14 (24.14)	35 (60.34)	9 (15.52)	58
SK	Poor	16 (25.40)	36 (57.14)	11 (17.46)	63
	Normal	25 (32.47)	40 (51.95)	12 (15.58)	77
	Good	10 (35.71)	13 (46.43)	5 (17.86)	28
Total		51 (30.36)	89 (52.98)	28 (16.67)	168

PI = Probabilistic Inference, SK = Statistical Knowlwdge

Table 11: Subjective prediction of the amount in the envelope under unknown uncertainty

(%) of respondents

	Forecasting Accuracy	Success	Failure	Total
Gender	Male	33 (40.24)	49 (59.76)	82
	Female	31 (36.05)	55 (63.95)	86
Major	Arts-related	2 (12.50)	14 (87.50)	16
	Business-related	10 (20.41)	39 (79.59)	49
	Math & Science	24 (61.54)	15 (38.46)	39
	Engineering & Tech	16 (69.57)	7 (30.43)	23
	Literature & Social Sci	5 (25.00)	15 (75.00)	20
	Others	11 (52.38)	1 (4.76)	21
Statistics	Yes	47 (52.81)	42 (47.19)	89
	No	17 (21.52)	62 (78.48)	79
PI	Weak	21 (19.09)	89 (80.91)	110
	Strong	43 (74.14)	15 (25.86)	58
SK	Poor	16 (25.40)	47 (74.60)	63
	Normal	27 (35.06)	50 (64.94)	77
	Good	21 (75.00)	7 (25.00)	28
Total		64 (38.10)	104 (61.90)	168

PI = Probabilistic Inference, SK = Statistical Knowlwdge

Table 12: Subjective forecast accuracy of the amount in the envelope under unknown uncertainty

OLS model for ∇WW

	Under Unknown Uncertainty							
	Maximized uncertainty				Informed uncertainty			
	Coefficient	S.E.	t	p-valued	Coefficient	S.E.	t	p-valued
Gender (Male)	0.0866	0.06 (0.06)	1.47	0.144 (0.166)	0.0699	0.07 (0.07)	1.06	0.291 (0.299)
Arts-related	- 0.0834	0.12 (0.09)	- 0.67	0.501 (0.342)	0.0128	0.11 (0.10)	0.11	0.911 (0.898)
Business-related	0.0705	0.10 (0.08)	0.72	0.471 (0.364)	- 0.0301	0.09 (0.07)	- 0.35	0.730 (0.675)
Math & Science related	0.0911	0.12 (0.11)	0.79	0.433 (0.390)	0.1697	0.10 (0.10)	1.63	0.100* (0.105)
Engineering & Technology	0.0276	0.13 (0.11)	0.21	0.831 (0.810)	0.2292	0.11 (0.12)	2.00	0.047** (0.064)
Literature & Social Science	0.0428	0.11 (0.10)	- 0.67	0.501 (0.656)	- 0.0283	0.10 (0.10)	- 0.27	0.785 (0.769)
Statistics	- 0.0663	0.07 (0.06)	- 1.02	0.309 (0.258)	0.2071	0.07 (0.07)	2.92	0.004*** (0.006)
Saving Accounts	- 0.0276	0.09 (0.08)	- 0.32	0.752 (0.727)	0.1521	0.08 (0.06)	1.98	0.050** (0.011)
Auto Loans	- 0.0254	0.08 (0.07)	- 0.36	0.722 (0.724)	- 0.0323	0.07 (0.06)	- 0.51	0.608 (0.574)
Residency (Urban)	- 0.0159	0.06 (0.06)	- 0.28	0.783 (0.786)	- 0.0051	0.05 (0.05)	- 0.10	0.920 (0.922)
Probabilistic inference	0.2957	0.15 (0.15)	1.88	0.062* (0.056)	0.3757	0.15 (0.16)	2.48	0.014** (0.020)
Statistical Knowledge	0.1355	0.14 (0.15)	1.02	0.311 (0.370)	0.3295	0.12 (0.15)	2.79	0.006*** (0.026)
Risk-Averse	0.0269	0.10 (0.08)	0.26	0.796 (0.749)	0.1345	0.07 (0.07)	1.85	0.067* (0.067)
Risk- Seeker	0.0643	0.08 (0.09)	0.81	0.420 (0.464)	0.0728	0.08 (0.08)	0.92	0.358 (0.362)
Overestimation	0.0437	0.08 (0.09)	0.55	0.581 (0.609)	- 0.0051	0.10 (0.09)	- 0.05	0.960 (0.955)
underestimation	- 0.1313	0.06 (0.06)	- 2.10	0.038** (0.021)	0.0629	0.07 (0.07)	0.84	0.402 (0.359)
Accuracy (Success)					0.2446	0.08 (0.08)	3.28	0.001*** (0.001)
Intercept	- 0.0502	0.13 (0.11)	- 0.37	0.710 (0.650)	- 0.6190	0.13 (0.11)	- 4.59	0.000*** (0.000)

Maximized Uncertainty: this is for the cases where subjects do not receive information under uncertainty.

Informed Uncertainty: this is for the cases where subjects receive information about the consequences of others under uncertainty.

The table presents OLS regressions for the changes in willingness to wait in percentage points.

Robust standard errors and p-value in parentheses. * $p < 0.1$, ** $p < 0.05$, *** $p < 0.01$.

Table 13: OLS regression for the changes in willingness to wait under unknown uncertainty
